# A metabolome and transcriptome survey to tap the dynamics of fruit prolonged shelf-life and improved quality within Greek tomato germplasm

**DOI:** 10.3389/fpls.2023.1267340

**Published:** 2023-09-25

**Authors:** Ifigeneia Mellidou, Athanasios Koukounaras, Sarah Frusciante, José L. Rambla, Efstathia Patelou, Symela Ntoanidou, Clara Pons, Stefanos Kostas, Konstantinos Nikoloudis, Antonio Granell, Gianfranco Diretto, Angelos K. Kanellis

**Affiliations:** ^1^ Institute of Plant Breeding and Genetic Resources, Hellenic Agricultural Organization – DEMETER, Thessaloniki, Greece; ^2^ Group of Biotechnology of Pharmaceutical Plants, Laboratory of Pharmacognosy, Department of Pharmaceutical Sciences, Aristotle University of Thessaloniki, Thessaloniki, Greece; ^3^ Department of Horticulture, Aristotle University of Thessaloniki, Thessaloniki, Greece; ^4^ Italian National Agency for New Technologies, Energy, and Sustainable Development (ENEA), Biotechnology Laboratory, Casaccia Research Center, Rome, Italy; ^5^ Instituto de Biología Molecular y Celular de Plantas (IBMCP), Consejo Superior de Investigaciones Científicas (CSIC), Universitat Politècnica de València, València, Spain; ^6^ Department of Biology, Biochemistry and Natural Sciences, Universitat Jaume I, Castellón de la Plana, Spain; ^7^ Instituto de Conservación y Mejora de la Agrodiversidad Valenciana (COMAV), Universitat Politècnica de València, València, Spain; ^8^ Agroindustrial Cooperative of Tympaki, Tympaki, Greece

**Keywords:** landraces, metabolomics, nutritional quality, ripening, shelf-life, storage, transcriptomics

## Abstract

**Introduction:**

Tomato is a high economic value crop worldwide with recognized nutritional properties and diverse postharvest potential. Nowadays, there is an emerging awareness about the exploitation and utilization of underutilized traditional germplasm in modern breeding programs. In this context, the existing diversity among Greek accessions in terms of their postharvest life and nutritional value remains largely unexplored.

**Methods:**

Herein, a detailed evaluation of 130 tomato Greek accessions for postharvest and nutritional characteristics was performed, using metabolomics and transcriptomics, leading to the selection of accessions with these interesting traits.

**Results:**

The results showed remarkable differences among tomato Greek accessions for overall ripening parameters (color, firmness) and weight loss. On the basis of their postharvest performance, a balance between short shelf life (SSL) and long shelf life (LSL) accessions was revealed. Metabolome analysis performed on 14 selected accessions with contrasting shelf-life potential identified a total of 206 phytonutrients and volatile compounds. In turn, transcriptome analysis in fruits from the best SSL and the best LSL accessions revealed remarkable differences in the expression profiles of transcripts involved in key metabolic pathways related to fruit quality and postharvest potential.

**Discussion:**

The pathways towards cell wall synthesis, polyamine synthesis, ABA catabolism, and steroidal alkaloids synthesis were mostly induced in the LSL accession, whereas those related to ethylene biosynthesis, cell wall degradation, isoprenoids, phenylpropanoids, ascorbic acid and aroma (*TomloxC*) were stimulated in the SSL accession. Overall, these data would provide valuable insights into the molecular mechanism towards enhancing shelf-life and improving flavor and aroma of modern tomato cultivars.

## 1 Introduction

Based on FAOSTAT database for 2020, tomato (*Solanum lycopersicum* L.) has an extremely high economic value importance worldwide, cultivated in over five million hectares with a production of over 186 million tons. The existing biodiversity within tomato genetic resources during its cultivation history is mainly the result of the progressive anthropogenic selection pressure (Pons et al., 2022). Through the long-term domestication process, farmer- and breeder-driven choices had narrowed down tomato genetic diversity, limiting their improvement potential. Nowadays, growers almost exclusively use tomato hybrids, focusing on yield performance and disease resilience, that comes usually at the expense of fruit taste, aroma, and phytonutrients. In Europe, and especially in Mediterranean countries, a remarkable phenotypic diversity related to fruit quality, aroma and taste attributes, as well as postharvest potential is evident, mostly because of the long cultivation history in the area, which therefore can be regarded as a secondary diversity hotspot (Mazzucato et al., 2008; Blanca et al., 2022). In the past few years, there were numerous genetic studies aiming to depict diversifications among the existing genetic pools in different South European countries (Cortés-Olmos et al., 2015; Sacco et al., 2015; Parisi et al., 2016; Blanca et al., 2022; Pons et al., 2022), some of them including Greek Gene Bank accessions.

Tomato is a typical climacteric fruit, that can be harvested either in early mature or full ripe stage depending on the market requirements, the distance until the retail market, or the season of the year. On the basis on their end-use, tomatoes can be classified as processing, fresh market, cherry, or traditional tomatoes, with the first three groups corresponding to breeding material characterized by the introgression of genes for disease resistance (Blanca et al., 2022). On the contrary, traditional varieties, including heirlooms, landrace and “vintage” germplasm, have not been widely incorporated into advanced breeding programs, presumably due to the lack of comprehensive genomic information within this traditional group. Thus, although several pre- and postharvest factors including cultivation practices, climate, or storage conditions have been widely studied (Conesa et al., 2014), it was only recently that the genomic effect has gained attention (Blanca et al., 2022; Pons et al., 2022), in order to unravel the history and domestication of the European traditional varieties. Notwithstanding the large genetic erosion with the gene pool present within modern varieties that has led to a significant drop-off in tomato aroma, some traditional varieties, with excellent taste and aroma properties, can be still found in local markets (Pons et al., 2022). Among them, some Greek varieties like “Tomataki Santorinis” are highly appreciated by consumers, being important for the local market (Koutsika-Sotiriou et al., 2016). However, the majority of this Greek traditional germplasm has been exploited for morphological diversity, heterogeneity and yield (Gonias et al, 2019; Mellidou et al., 2020), without any available information about their nutritional value and postharvest life performance. In this frame, the characterization of this traditional germplasm for its future exploitation can provide a valuable reservoir of genes not only to restore tomato taste and aroma in the commercial varieties, but also due to its great adaptability to local environmental and farming conditions.

Despite these genetic studies aiming to unveil the diversification history of the European traditional tomato varieties, a comprehensive study on its fruit composition, nutritional value and flavor attributes is still lacking. Globally, tomato is among the main consumed crops essential for the human diet, containing many healthy and beneficial biologically active compounds, including lycopene, vitamin C and E, and flavonoids (Klee, 2010; Ilahy et al., 2019). The majority of these metabolic routes have been well-characterized in tomato fruit, with a plethora of transgenic lines enriched in these compounds (Verhoeyen et al., 2002; Bulley et al., 2012; Koukounaras et al., 2022). At the same time, tomato fruit contains high amounts of steroidal alkaloids and glycoalkaloids, such as α-tomatine, that are toxic for humans, thus considered as antinutritional factors (Zhao et al., 2023). A wide group of genes related to glycoalkaloid metabolism (*GAME*) has been previously demonstrated to participate in glycoalkaloid synthesis, whereas through the process of ripening and a series of hydroxylation and glycosylation reactions, these genes convert tomatine to the non-toxic and non-bitter esculeoside A (Cárdenas et al., 2016) that also possess some health benefits (Nohara et al., 2010).

Along with these important phytonutrients that are appreciated by consumers, tomato flavor greatly affects consumer acceptance and preferences, re-directing breeders’ goal from yield-oriented towards nutritional- and flavor-oriented strategies, regenerating the necessity of exploring the genetics underlying tomato flavor (Wang et al., 2023). Tomato’s unique flavor arises from the balance between sugars (glucose and fructose), acids (citrate, malate, ascorbate and glutamate) and volatile organic compounds (VOCs), constituting of a huge heterogeneous group of diverse molecules (Colantonio et al., 2022; Kaur et al., 2023). The loss of positive contributors to fruit aroma has been previously associated to linkage drag with other commercial traits such as fruit weight and disease resistance (Li et al., 2022a). Among the nearly 400 volatile compounds identified in tomato fruit and derived from essential phytonutrients, such as apocarotenoids, and derivatives of branched amino-acids, lipids/fatty acids, sulfur, phenylalanine and phenylpropanoid, only a small fraction of them are regarded as important contributors to the typical aroma of fresh tomato (Tieman et al., 2017). These include (*Z*)-3-hexenal, β-ionone, hexanal, β-damascenone, 1-penten-3-one, 3-methylbutanal, (*E*)-2-hexenal, (*Z*)-3-hexenol, 6-methyl-5-hepten-2-one, methyl salicylate and 2-isobutylthiazole, with the rest contributing as background notes (Ilahy et al., 2019). The interaction of these compounds released sensorial odors described as “sweet”, “smoky”, or “fruity” (Martina et al., 2021). The dissatisfaction of consumers concerning the loss of tomato flavor in modern fresh market tomatoes is well-documented (Tieman et al., 2017), but breeding for enriched tomato flavor has proven to be a difficult task due to complex interacting metabolic networks as well as the strong environmental effect. However, the progression of fast-evolving analytical techniques and data mining tools for the accurate determination of “flavor-omics” (Bineau et al., 2022), along with the functional characterization of hub genes involved in these pathways, may open new avenues for plant breeding towards the biofortification of tomatoes (Alseekh et al., 2021). Nonetheless, there is still a long way towards the development of the “perfect” tomato, as well as an emerging need to exploit all the available germplasm collections to shed light into these multifaceted interactions.

In this study, a total of 130 accessions of tomato from the Greek Gene Bank (ELGO-Demeter) were initially characterized for fruit traits and postharvest performance. In turn, fruit metabolome of a selection of seven long shelf-life (LSL) and seven short shelf-life (SSL) varieties was exploited to identify differentially accumulated metabolites (DAMs) related to fruit quality, taste and flavor, between groups. As a further step, fruit transcriptomes of the best LSL and the best SSL genotypes, substantially differing in ethylene production, were investigated to expand our understanding on the relationships between the transcriptome and the postharvest shelf-life attributes. The knowledge produced herein can be valuable for future functional studies, and can assist breeders to improve decision-making towards the development of the “perfect” tomato.

## 2 Materials and methods

### 2.1 Plant material and growth conditions

The experiment was conducted in Tympaki, Crete Island, Greece, in a non-heated plastic greenhouse. The establishment of the crop was done according to the usual cultural practices. At the time that fruit were at the required maturity stage (see below), they were harvested and transferred within 24h to the facilities of Aristotle University of Thessaloniki for evaluation. During the 1^st^ year of the experiment, 130 accessions from the Greek Gene Bank (ELGO-Demeter) with five plants per accession were cultivated and evaluated (**Supplementary Figure 1**), as described below. In the 2^nd^ year of the experiment, 14 accessions selected based on the results of the previous year with 10 plants per accession were cultivated and evaluated.

### 2.2 Postharvest evaluation

During the 1^st^ year of the experiments, due to the absence of prior knowledge about the postharvest behavior of the accessions, both SSL and LSL protocols were employed in order to properly classify accessions based on their postharvest characteristics. These two protocols were commonly applied in all postharvest experiments performed by the EU-TRADITOM partners. In particular, for the SSL protocol (Protocol A), fruits at the breaker stage were harvested and stored at 12 °C for 10 days, and then transferred to 20 °C for 3 days. Fruit weight, fruit color with the use of a digital colorimeter (CR-400 Chroma Meter, Konica Minolta Inc., Tokyo, Japan), as well as firmness with durometer (Turoni, Italy), were recorded in all fruits during the cold storage period (CS), and during shelf life (SL), as well as both periods (total). In particular, firmness loss (FL) was expressed as the average of the % of firmness loss (FL) and is calculated as FL=100-(F_t_*100/F_0_). Where F_t_ is the measured firmness at each time and F_0_, the measured firmness at the reference time. Color Index change (ΔCa/b or CI) was expressed as the average of ratio a/b increment in relation to the reference time. Weight loss was expressed as % of the initial value. On the other hand, for the LSL protocol (Protocol B), fruits at the red stage were harvested and stored at 20 °C (Protocol B). Once per week, red fruits were evaluated for weight loss, firmness, color, as described above, as well as for decay. As a result of the 1^st^ year experimentation, the accessions were classified as short self life (SSL: shelf life < 1 week), as medium shelf life (MSL: shelf life < 2 weeks) or as long shelf life (LSL: shelf life > 2 weeks). During the 2^nd^ year of experimentation, the evaluation protocol A was applied for SSL accessions, while protocol B for the LSL accessions.

The ethylene production rate was measured during the 2^nd^ year by injecting a gas sample, 1 mL each, which was taken from the exit tube of a jar with fruit inside, into a gas chromatograph (model Varian 3300, Varian Instruments, Walnut Creek, CA) equipped with a flame ionization detector, essentially as previously described (Koukounaras et al., 2022). To identify significant differences in ethylene production between SSL and LSL genotypes, Tukey’s test with significant level P ≤ 0.05 was performed using SPSS 27 (IBM, USA).

### 2.3 Non-volatile metabolomic profiling

Non-polar (NP) and semi polar (SP) metabolites were extracted from, respectively, 3 and 10 mg of freeze-dried tomatoes, harvested at the red stage, for a total of three replicates for each of the seven SSL and the seven LSL genotypes. Liquid chromatography coupled to high-resolution mass spectrometry (HPLC-APCI/ESI-HRMS) conditions were as previously reported for NP (Teixeira et al., 2023) and SP (Dono et al., 2022) metabolome, respectively. Metabolites were identified through the comparison with authentic standards or MS/MS spectra when available and on the basis of the accurate masses obtained from the Pubchem database (http://pubchem.ncbi.nlm.nih.gov/) for native compounds or from the Metabolomics Fiehn Lab Mass Spectrometry Adduct Calculator (http://fiehnlab.ucdavis.edu/staff/kind/Metabolomics/MS-Adduct-Calculator/) for adducts. Data were quantified as fold internal standard levels: alfa-tocopherol acetate (Cat. N. 47786, Sigma Aldrich) has been used in case of NP metabolites and daidzein (Cat. N.16587-10MG, Supelco) for SP metabolites.

### 2.4 Determination of volatile compounds

Headspace solid phase microextraction (HS-SPME) was used for the capture of volatile compounds, which were then separated and detected by means of gas chromatography coupled to mass spectrometry (GC/MS), similarly as described in Rambla et al. (2017). The pericarp of red ripe tomato fruits from seven SSL and seven LSL genotypes, was excised, immediately frozen in liquid nitrogen, milled to a fine powder under cryogenic conditions and stored at -80°C until analysis. Three biological replicates were analyzed, each consisting a mixture of two or three fruits.

Five hundred milligrams of the tomato samples were placed in a 15 mL glass vial and incubated in a water bath for 10 min at 37°C. Five hundred mL of an EDTA 100 mM, pH 7.5 solution and 1.1 g of CaCl_2_.2H_2_O were added, gently mixed and sonicated for 5 min. One mL of the resulting paste was transferred to a 10 mL screw cap headspace vial with silicon/PTFE septum and analyzed within 10 hours. Volatile compounds in the headspace were extracted by a 65 µm PDMS/DVB SPME fiber (Supelco). Volatile extraction was performed automatically with a CombiPAL autosampler (CTC Analytics). Vials were first incubated at 50°C for 10 min with 500 rpm agitation. The fiber was then exposed for 20 min to the vial headspace under the same agitation and temperature conditions. Desorption was performed at 250°C during 1 min in a 6890N gas chromatograph (Agilent) in splitless mode. After desorption, the fiber was cleaned in an SPME fiber conditioning station (CTC Analytics) at 250°C for 5 min under a helium flow to prevent cross contamination. Chromatography was performed on a DB-5ms (60 m, 0.25 mm, 1 µm) capillary column (J&W) with 1.2 mL/min constant helium flow. Temperatures of the GC interface and MS source were 260°C and 230°C, respectively. Oven programming conditions were 40°C for 2 min, 5°C/min ramp until 250°C, and a final hold at 250°C for 5 min. Data was recorded in a 5975B mass spectrometer (Agilent) in the 35-250 m/z range at 6.2 scans/s, with 70 eV electronic impact ionization. Chromatograms were processed by means of MassHunter version 10.1 (Agilent). Identification of compounds was based on comparison of both retention time and mass spectrum with those of pure standards (Merck). For quantitation, the extracted ion chromatogram for one specific ion was integrated for each compound. The criteria for ion selection were specificity and high signal-to-noise ratio. An admixture reference sample was prepared for each season by thoroughly mixing equal amounts of each sample. A 500 mg aliquot of the admixture was analyzed regularly (one admixture for every ten samples) and processed as any sample as part of the injection series. This admixture was used as a reference to normalize for temporal variation and fiber aging. The normalized results for a sample are expressed as the ratio of the abundance of each compound in that particular sample to those in the reference admixture.

Finally, the identified metabolites were subjected to Principal Component Analysis. Heatmaps and hierarchical clustering of the identified metabolites were constructed using Clustvis online tool (Metsalu and Vilo, 2015). The software package “corrplot” in R was used to calculate the Pearson correlation coefficient of metabolites of the 14 genotypes (Wei and Simko, 2017). To identify significant differences within the metabolomic data between the best SSL and the best LSL genotypes, Student’s T-test at 0.05 level was performed using SPSS 27 (IBM, USA).

### 2.5 RNA isolation and sequencing

Fruits of the best LSL (TRTH1620) and the best SSL (TRTH2510) genotypes at the red stage of ripening were used for total RNA extraction using Spectrum Plant Total RNA kit (Sigma-Aldrich, St. Louis, MO, USA). Three biological replicates were used per genotype. Quantification and qualification of the extracted RNA was checked using Nanodrop 2000 (NanoDrop Technologies, Wilmington, DE, USA) and 1% agarose gel electrophoresis, or Agilent 4200 TapeStation System (Agilent, CA, USA), respectively. Six RNA samples were sent to the Beijing Genome Institute (BGI, Shenzhen, China) for sequencing (BGI, Shenzhen, China). Raw data were deposited in the National Centre for Biotechnology Information (NCBI) Sequence Read Archive (SRA) under BioProject accession number: PRJNA991925.

### 2.6 Transcriptome data analysis

As a first step, BBDuk (Decontamination Using Kmers) was employed to trim and filter the raw sequences, in Geneious Prime v2021.1.1. Adapters were trimmed on paired read overhangs, with minimum overlap of 20, while short reads with minimum length of 20 were discarded. The high quality paired-end reads were then aligned to the tomato reference genome (Hosmani et al., 2019) using Geneious Prime Mapper with high sensitivity, essentially as previously described. Afterwards, the mapped reads for each transcript were generated using the Calculate Expression Levels feature in Geneious Prime, to calculate fragments per kilobase for exon model per million mapped reads (FPKM). Differentially Expressed Genes (DEGs) between TRTH1620 and TRTH2510 were calculated using DESeq2 package in R (Love et al., 2014). Transcripts were screened based on adjusted p ≤ 0.01 and |Log2(fold-change, FC)| ≥ 2, to identify the most significant DEGs. Finally, Gene Ontology (GO) and KEGG enrichment analysis of DEGs was investigated using clusterProfiler in R studio (Yu et al., 2012), to elucidate the functional representation of genotypic differences.

### 2.7 RT-qPCR verification

Quantitative real-time PCR (qPCR) analysis was performed to verify the RNAseq results, using the same total RNA per sample. Based on transcriptomic data, the expression levels of eleven (11) genes from key metabolic pathways during tomato ripening were investigated. The cDNA was synthesized with the Superscript^®^ III Reverse Transcriptase kit (Invitrogen), according to manufacturer’s instructions. Primers were developed using the genomic data of the two genotypes (Pons et al., 2022). The internal standard used in the qPCR analysis was the endogenous tomato gene CAC (clathrin adaptor complexes medium subunit) (Mellidou et al., 2012). Each sample was represented by three biological replicates and all qPCR analyses were performed in duplicates. The qPCR reactions were performed in a 20 μl reaction volume containing 0.2 ng cDNA, 0.1 pmol each primer, 1 × KAPA SYBR FAST Master Mix (KAPABIOSYSTEMS, Boston, U.S.A.) and sterile, nuclease free H2O. The amplification was conducted at 95 °C for 4 min, followed by 6 cycles of 95 °C for 35 s, T annealing, 57 °C for 30 s and 72 °C for 20 s, and in turn by 44 cycles of 95 °C for 25 s, T annealing, 57 °C for 20 s, 72 °C for 15 s, and plate read at 80 °C. To identify the PCR products a melting curve was performed from 65 °C to 95 °C with readings every 0.1 °C and a 10-s hold between observations. Relative quantification and statistical analyses were done using the comparative CT (ΔΔCT) method (Schmittgen and Livak, 2008), with the DataAssist™ v3.0 data analysis tool. Relative quantification of gene expression was carried out in respect to differences observed between TRTH1620 and TRTH2510. The primers used for RT-qPCR are listed in **Supplementary Table 1**.

## 3 Results

### 3.1 Post-harvest dynamics of the tomato accessions

**Figure 1:**
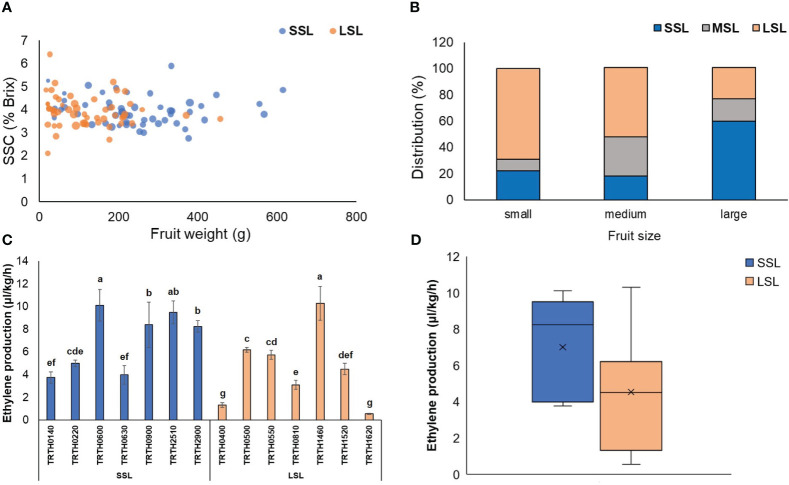
**(A)** 4-D bubble plot analysis displaying the relationship between fruit weight, SSC (°Brix), fruit firmness (%) and postharvest characterization. The x-axis represents fruit weight (g), the y-axis represents °Brix, the bubble size represents firmness, and the bubble color the postharvest characterization. **(B)** Distribution (%) of the 130 tomato accessions classified as SSL, MSL and LSL, based on their fruit size. **(C)** Ethylene production rate (μl kg^-1^ h^-1^) of seven (7) SSL and seven (7) LSL accessions, determined at the red stage of ripening. Different letters indicate statistically significant differences based on Tukey’s test (P < 0.05). **(D)** Boxplots of the average ethylene production (μl kg^-1^ h^-1^) determined in the seven (7) SSL and the seven (7) LSL accessions. SSL, short shelf-life; MSL, medium shelf-life; LSL, long shelf-life.

Among the 130 tomato Greek accessions, there were highly significant differences (*P* < 0.01 or <0.001) for overall ripening parameters, weight loss as well as fruit weight (**Supplementary Table 2**). The average firmness loss rate during the whole storage period was found to be 3.66%, ranging widely from 1.49 to 6.45%. Similarly, for color index change the mean value was 0.093, the lowest and the maxim value were 0.014 and 0.144, respectively. Classification of accessions based on the postharvest performance showed a balance between SSL and LSL (**Figure 1A**), with a portion of 42.3 and 40.8%, respectively. Fruit size had a significant effect over the classification of the accessions to a shelf-life category. In particular, for small-fruited accessions, the postharvest performance was relatively good (69% of the accessions characterized as LSL, and only about 22% as SSL). Large-fruited accessions had the opposite behavior (60% of the accessions characterized as SSL and only about 24% as SSL). Accessions with medium fruit size showed an intermediate performance and classification as MSL (**Figure 1B**).

Out of the 130 accessions, seven LSL and seven SSL varieties were selected based on the results of the 1^st^ year in order to repeat post-harvest phenotyping during the 2^nd^ year. Heatmaps for SSL accessions showed that TRTH0140 and TRTH0220 had the best postharvest performance, exhibiting extremely slow color change, softening and low weight loss for the whole storage period, followed by TRTH2510 and TRTH0900 that also performed well (**Supplementary Figure 2A**). By contrast, TRTH0630 exhibited the worst postharvest performance. Accordingly, the best performing accession from the LSL group was TRTH1520, followed by TRTH1620, with decay rates after 4 weeks storage of 5% and 11%, respectively, whereas a number of accessions showed extremely low storability (**Supplementary Figure 2B**). All the above findings were generally in line with the ethylene production rate of the fruits, with SSL accessions exhibiting in average the highest rates (**Figures 1C, D**), and those classified as LSL (especially TRTH1620 and TRTH0400) low ethylene production. Nonetheless, there were some LSL accessions (TRTH1460, TRTH0500, TRTH0550) that exerted high ethylene production, at similar levels to SSL accessions. In future studies, this observation might be of interest in understanding how i.e. TRTH1460 exhibits high ethylene production rates, yet being LSL.

### 3.2 Metabolomic analysis of the differences in phytonutrients and volatiles within the collection

To explore the differences in flavor and taste compounds among the 14 selected Greek tomato accessions, a metabolomic comparison including NP and SP metabolites, as well as volatiles, of seven LSL and seven SSL cultivars was performed. A total of 206 metabolic compounds were identified, among them, 17 amino acids, 13 organic acids, 17 carotenoids, 36 polyphenolic compounds, and 77 volatiles (**Supplementary Table 3**). Cluster analysis of metabolite concentration indicated that all biological replicates for the same accession were grouped together, indicating a high reliability and reproducibility of the resulting metabolome data (**Supplementary Figure 3**).

**Figure 2:**
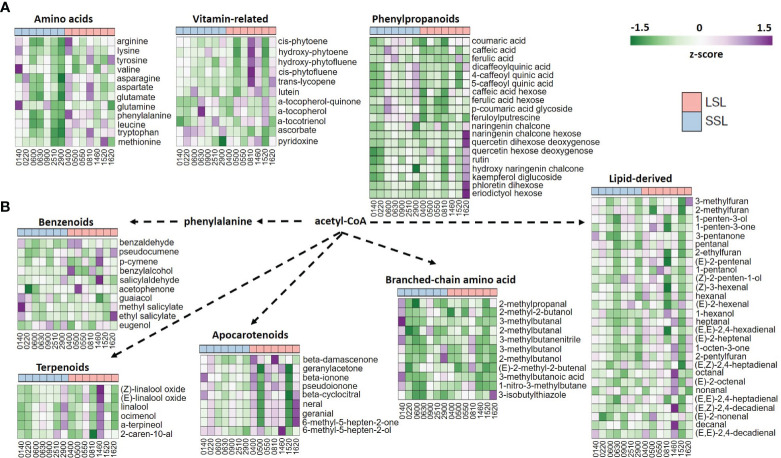
Heatmaps of the metabolomic profiles of the seven SSL and the seven LSL genotypes, with regard to amino acids, vitamins and phenylpropanoid abundance **(A)**, as well as to volatiles **(B)**. Mean values represent the average of three biological replicated, and are normalized based on z-score.

Principal component analysis (PCA) was performed, PC1 and PC2 explaining 36.3% and 17.3% of total variance, respectively (**Supplementary Figure 4**). Accessions in the first component were separated mainly by total firmness loss after postharvest, being those with the highest firmness loss. The second component, mostly separated the LSL accession TRTH1620 from the rest of accessions (**Figure 2**; **Supplementary Figure 3**). The genotype TRTH1620 had a distinguished metabolome profile, being particularly rich in the majority of flavonoid compounds, as well as in esculeoside A, a steroidal alkaloid glycoside, possessing potential health benefits for humans (Huang et al., 2021), as will be discussed below.

Within metabolome data according to our experimental conditions, the most abundant sugar was glucose, whereas pyruvic and lactic acids were the top organic acids (**Supplementary Table 3**). Among the amino acids, tomato fruits were rich in glutamate, representing the umami perception, and phenylalanine, followed by leucine/isoleucine and tryptophan. With regard to postharvest potential, it was evident that LSL genotypes accumulated more amino acids than SSL genotypes, except for TRTH0140, which belongs to the SSL group (**Figure 2**). Similarly, the most abundant carotenoids were 15-cis-phytoene, all-trans-lycopene and 9,15-cis-phytofluene, whereas vitamin E (α-tocopherol), vitamin C (ascorbic acid), and vitamin B6 (pyridoxine) were also abundant, yet with remarkable differences between the different varieties. In particular, LSL genotypes seemed to accumulate more carotenoids than SSL ones, whereas SSL genotypes had more tocopherols and ascorbic acid than LSL ones. Notably, the highest pyridoxine levels were measured in TRTH0220 (SSL) and TRTH1460 (LSL). Among the phenylpropanoids, 4-caffeoyl-quinic acid, caffeic acid hexose and naringenin chalcone were the most represented compounds, with LSL genotypes being richer in flavonoids, and the SSL genotypes in hydroxycinnamic acids, with only a few exceptions.

The 77 volatile compounds were grouped as apocarotenoids, benzenoids, branched-chain amino acid related, phenylalanine-derived, sulfur compounds, terpenoids, esters and lipid-derived, that contained nearly half of the identified VOCs (**Supplementary Table 3**). PCA analysis and heatmap depicted a remarkable variability in volatile compounds among the 14 accessions, however they also indicated that VOC did not seem to correlate with postharvest properties (**Supplementary Figure 5**). For example, TRTH0140 (SSL) was rich in VOCs derived from the benzenoid and branched-chain amino acid pathways, but so did the LSL varieties TRTH1460 and TRTH0810, respectively. Furthermore, apocarotenoids such as geranial, neral, and 6-methyl-5-hepten-2-one were highly abundant in TRTH1620, and to a lesser extent in TRTH1460, TRTH0550 and TRTH0810, all from the LSL group, but not in other LSL varieties such as TRTH0500 and TRTH1520, suggesting the complexity of the routes involved in tomato aroma, flavor and postharvest attributes. The LSL variety TRTH1460 also contained the highest amounts of terpenoids and lipid-derived VOCs, which also correlated with the highest ethylene accumulation determined within the collection (**Figure 1C**). In this LSL genotype, an interesting correlation between apocarotenoid VOCs that are related to precursors localized in plastids and increased during ripening, and lipid-derived VOCs that are linked to precursors of membrane fatty acids, was evident, in line with previous reports (Colantonio et al., 2022). Additionally, TRTH0500 and TRTH0550, both from the LSL group, were rich in 1-penten-3-one, an important compound for tomato flavor. The LSL variety TRTH0400 contained high amounts of the umami-related influencers, 2-phenylethanol and 1-nitro-2-phenylethane, from the phenylalanine-derived pathway. The lipid-derived VOC (*E*)-2-pentenal, previously identified as important contributor to flavor enhancement and umami, was highly accumulated in the LSL varieties, TRTH0400, TRTH0500 and TRTH0550.

By comparing the average values of SSL and LSL varieties, a total of 15 metabolites (amino acids, organic acids, carotenoids, hydroxycinnamic acids and VOCs) were significantly differentially accumulated in the fruits with the contrasting postharvest properties (**Table 1**; **Supplementary Table 4**). Among them, the amino acids phenylalanine, leucine and tryptophan, the organic acids, adipate, glutarate and itaconate, as well as 1-octen-3-one (lipid-derived VOC), were up-regulated in the LSL varieties. By contrast, ascorbic acid (AsA), hydroxyproline, 9-cis-violaxanthin, two hydroxycinnamic acids (ferulic acid hexose), N-feruloylputrescine, and three ester-related VOCs (2-methylpropyl acetate, 3-methylbutyl acetate and 2-methylbutyl acetate) were up-regulated in the SSL varieties. The small number of significant correlations found between the 206 compounds and postharvest potential within this germplasm collection, clearly suggest that promising germplasm with excellent flavor and taste can also be detected among varieties with prolonged storability.

**Table 1:** Differentially accumulated metabolites between SSL and LSL based on Student’s t-test.

		Average content			
Group	Metabolite	SSL	LSL	log2FC (LSL/SSL)	sig.
amino acids	phenylalanine	104.2 ± 22.0	193.8 ± 30.8	0.27	*
	leucine/isoleucine	46.77 ± 8.65	73.18 ± 7.37	0.19	*
	tryptophan	15.38 ± 2.48	26.82 ± 2.93	0.24	*
	hydroxyproline	0.10 ± 0.01	0.08 ± 0.01	-0.09	*
organic acids and derivatives	ascorbic acid	2.67 ± 0.55	1.59 ± 0.26	-0.22	**
	adipic acid	0.48 ± 0.08	0.73 ± 0.07	0.18	*
	glutaric acid	1.09 ± 0.02	1.30 ± 0.05	0.07	**
	itaconic acid	0.56 ± 0.03	0.73 ± 0.03	0.11	**
carotenoids	9-cis-violaxanthin	0.014 ± 0.001	0.008 ± 0.002	-0.27	*
hydroxycinnamic acids and derivatives	Ferulic acid hexose	7.66 ± 1.19	4.31 ± 0.84	-0.25	*
	N-Feruloylputrescine	5.51 ± 0.76	2.24 ± 0.49	-0.39	**
VOCs - esters	2-methylpropyl acetate	1.20 ± 0.14	0.80 ± 0.11	-0.18	*
	3-methylbutyl acetate	1.25 ± 0.16	0.75 ± 0.11	-0.22	*
	2-methylbutyl acetate	1.47 ± 0.27	0.53 ± 0.10	-0.44	*
VOCs - lipids	1-octen-3-one	0.87 ± 0.03	1.13 ± 0.08	0.11	*

Values represent mean values ± SE.

Pearson correlation coefficient for all the abundant metabolites (36 compounds and 60 VOCs) was calculated (**Supplementary Table 5**), with those having R^2^> 0.8 being illustrated in **Supplementary Figure 6**. The most significant correlations in our sample set were identified between rutin and kaempferol diglucoside (R^2 =^ 0.99), aspartate and glutamate (R^2 =^ 0.95), as well as glutamate and pyruvate (R^2 =^ 0.97). Other important correlations included various amino acids with each other (positive correlations), or amino acids with phenolic compounds (negative correlations). Similarly, regarding VOCs, neral and geranial (R^2 =^ 0.99) or 6-methyl-5-hepten-2-one (R^2 =^ 0.99), 2-phenylethanol and 1-nitro-2-phyenylethane (R^2 =^ 0.97), α-terpineol and methional (R^2 =^ 0.98) or linalool (R^2 =^ 0.96), (*E*)-2-heptenal and hexanal (R^2 =^ 0.97), as well as octenal and heptanal (R^2 =^ 0.96) were significantly correlated.

**Figure 3:**
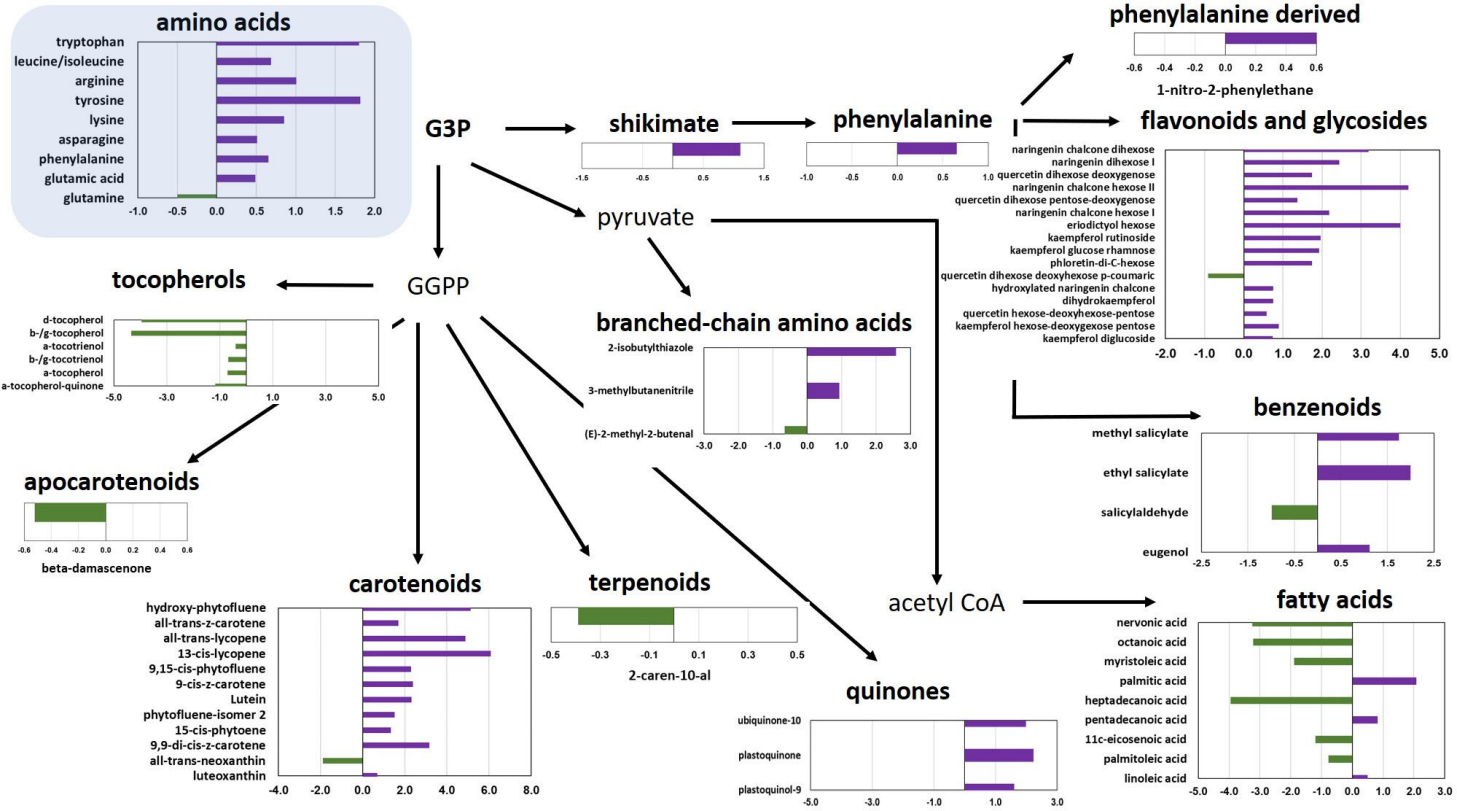
Significantly differentially accumulated metabolites over-accumulated in LSL (TRTH1620; purple) or in SSL (TRTH2510; green), with p< 0.05. Values represent log2 fold change in the LSL/SSL genotype.

As a further step, the metabolomic data of the best LSL (TRTH1620) and the best SSL genotype (TRTH2510) were utilized to identify the differentially accumulated metabolites (DAMs), with p<0.05 (**Figure 3**; **Supplementary Table 6**). Among the 93 compounds, there were a total of 13 VOCs from different pathways, 16 flavonoids, nine amino acids, and 12 carotenoids, of which 66 were up-regulated in the LSL and only 27 in the SSL genotype. Overall, individual amino acids were more abundant in the LSL genotype. Similarly, the shikimate pathway proceeding towards flavonoids was enhanced in TRTH1620, whereas the pathway from glyceraldehyde 3-phosphate towards the production of fatty acids was increased in TRTH2510. Regarding isoprenoid biosynthetic pathways, carotenoids and quinones were more abundant in the LSL genotype, while tocopherols in the SSL genotype. Other metabolites that were over-accumulated in the LSL genotype included esculeoside A and hydroxytomatine I (approximately 4-fold higher). The greatest differences were observed for all-trans-lycopene (carotenoids), naringenin chalcone hexose II and eriodictyol hexose (flavonoids and glycosides), as well as esculeoside B. With regard to VOCs that were differentially accumulated in the two genotypes, it was evident that one apocarotenoid (β-damascenone), one terpenoid (2-caren-10-al), one branched amino acid related (2-methyl-2-butenal), and one benzenoid related (salicylaldehyde) were significantly higher in the SSL genotype (**Figure 3**). By contrast, a phenylalanine-derived, three benzenoid-related, and two branched amino acid related, were more abundant in the LSL genotype. Among the VOCs, salicylaldehyde was the top differentially accumulated VOC in TRTH2510 (2-fold), whereas 2-isobutylthiazole and 1-nitro-2-phenylethane were the top differentially accumulated metabolites in TRTH1620 (6- and 10-fold, respectively).

### 3.3 Transcriptome sequencing, clustering and enrichment

**Figure 4:**
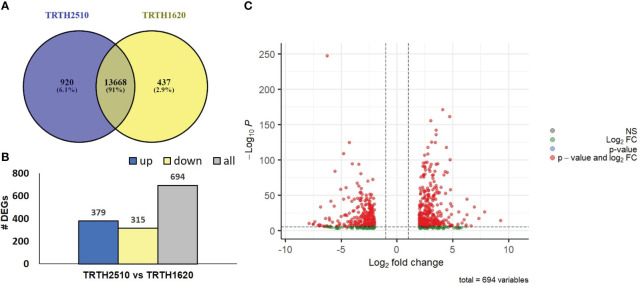
**(A)** Venn diagram of the expressed transcripts with FPKM>1 highlighting commonly and uniquely expressed genes in the fruits of TRTH2510 (SSL) and TRTH1620 (LSL); **(B)** The number of Differentially Expressed Genes (DEGs) representing up- and down-regulated transcripts of fruits in TRTH1620 (LSL) compared to TRTH2510 (SSL); **(C)** Volcano plot of DEGs in TRTH1620 (LSL) compared to TRTH2510 (SSL) based on p-values and log2 fold change (FC) of the transformed FPKM values. NS: non-significant.

As a further step towards exploring pathways contributing to fruit quality and postharvest potential, transcriptome analysis was performed in fruits from the best SSL, TRTH2510 and the best LSL, TRTH1620) accessions, producing high and low ethylene levels, respectively. A total of 524.7 M with an average of 87.4 M clean reads were generated for the six cDNA libraries (**Supplementary Table 7**). The clean reads had an average total mapping ratio of 95.6%. Total transcripts with FPKM>1, averaged from the three biological replicates, varied from 14105 in TRTH1620 to 14588 in TRTH2510 fruits (**Supplementary Table 8**). The abscisic stress-ripening protein 1 (ID543574) had the highest FPKM values in both genotypes. However, in LSL, it was followed by dehydrin (ID101253585), whereas in the SSL, by the metallocarboxypeptidase inhibitor TCMP-2 (ID543980) and a polygalacturonase (*PG*) precursor (ID544051). Commonly expressed transcripts accounted for 91% of the total expressed genes with FPKM >1, whereas the uniquely expressed transcripts ranged from 437 in LSL (2.9%) to 920 in SSL (6.1%) (**Figure 4A**).

DEGs were defined using │log2(FC) > 2│and corrected (p values ≤ 0.01). In total, 379 and 315 DEGs were up- or down-regulated, respectively, in fruits obtained from TRTH1620 compared to TRTH2510 (**Figure 4B**; **Supplementary Table 9**), highlighting a series of changes in their gene expression profiles that could be associated with short- or long-shelf-life regulatory points. The list of DEGs is also depicted in a volcano plot based on log2 transformed FPKM values (**Figure 4C**), underlying the fact that the majority of DEGs were up-regulated in fruits of the LSL line. Among the top up-regulated transcripts in the LSL genotype, there were two homologs of glutathione-S-transferases (LOC101253021, LOC101257702, LOC101244300), whereas in the SSL genotype, there were a carbonyl reductase [NADPH] (LOC101244566), related to folate biosynthesis, and *PG* (LOC101246713) (**Supplementary Table 9**).

Enrichment analysis of GO annotations of DEGs between TRTH1620 and TRTH2510 returned 204 GO terms, classified into the three GO classes (BP: Biological Process; MF: Molecular Function; CC: Cellular Component). Among them, 99 and 105 GO annotations were enriched in the LSL and the SSL genotypes, respectively (**Supplementary Figure 7**). In the LSL genotype, the most significant enrichment terms in BP category were carbohydrate metabolic process (GO:0005975), cellular glucan metabolic process (GO:0006073), and cellular polysaccharide metabolic process (GO:0044264), whilst in the MF category, there were nucleic acid binding transcription factor activity (GO:0001071) and transcription factor activity, sequence-specific DNA binding (GO:0003700). Transcripts from the extracellular region (GO:0005576) and the apoplast (GO:0048046) were also enriched. Accordingly, in the SSL genotype, the most significant enrichment terms were transferase activity, transferring acyl groups other than amino-acyl groups (GO:0016747) and pyridoxal phosphate binding (GO:0030170), as well as small molecule metabolic process (GO:0044281), oxoacid metabolic process (GO:0043436) and organic acid metabolic process (GO:0006082). As a further step, the DEGs were also examined against the KEGG database to identify active biological pathways between the two contrasting genotypes. However, no KEGG pathways were found to be significantly enriched in the LSL genotype, whilst in the SSL genotype, the most significant enriched pathway was phenylalanine metabolism (sly00360) that is associated with phenylpropanoid pathway.

To validate these DEGs between TRTH1620 and TRTH2510, several key genes from metabolic pathways were selected, and their expression levels were analyzed using RT-qPCR. For all the transcripts, their expression levels were in agreement with those of the RNA-Seq (R^2 =^ 90.3) (**Supplementary Figure 8**), suggesting the reliability of the RNA-seq dataset for further exploitation. In particular, it was verified that the transcript levels of ethylene biosynthetic genes such as 1-aminocyclopropane-1-carboxylate synthases (*ACS2*, *ACS4*), and 1-aminocyclopropane-1-carboxylate oxidase (*ACO1*) were induced in SSL (4.1-, 66- and 3.4-fold higher than LSL) (**Supplementary Figure 9**). Similarly, genes related to AsA biosynthesis (GDP-_L_-galactose phosphorylase, *GGP*), carotenoids biosynthesis (phytoene synthase, *PSY1*), cell wall synthesis (*PG2*), phenylpropanoid pathway (1-Deoxy-D-xylulose 5-phosphate synthase, *DXS*; chalcone synthase*, CHS1*; geranylgeranyl pyrophosphate synthase*, GGPS2*), and lipid-derived volatiles (lipoxygenase, *TomloxC*) were remarkably up-regulated in SSL. These genes represent the rate-liming steps in the related pathways.

### 3.4 Differentially expressed genes related to fruit quality, nutrient value and aroma

By considering DEGs as those with p_adj_ ≤ 0.01, with no strict log2FC criterion, we ended up with a list of genes that may be able to explain the differences in the quality and metabolome reported herein between TRTH1620 (LSL) and TRTH2510 (SSL) (**Supplementary Table 10**). Several genes related to cell wall were differentially expressed between the two genotypes. In agreement with postharvest potential of the two contrasting genotypes, 21 cell-wall biosynthetic or expansion genes were up-regulated only in LSL, among them there were cellulose synthases (LOC101247596, LOC101249747, LOC101267858, LOC101260024), prolyl 4-hydroxylases (LOC101246711, LOC101262682), xyloglucan galactosyltransferases (LOC101245582, LOC101263331), a xyloglycan endo-transglycosylase (*tXET-B2*), xyloglucan endotransglucosylase/hydrolases (*XTH3*, LOC101255416, LOC101258345, LOC543511) and a xyloglucan 6-xylosyltransferase (LOC101263199). By contrast, 14 transcripts related to cell-wall degradation were up-regulated in SSL and suppressed in LSL, including *PG2* (LOC101246713), beta-galactosidases (*TBG1*, *TBG3*), and several pectinesterases (*PE1*, *PME2.1*, LOC101268487, LOC101254166, LOC101258356).

In addition, it was evident that the majority of key ethylene biosynthetic genes, namely *ACS2*, *ACS4*, *ACO1*, *ACO3*, *ACO4* were up-regulated in SSL, (**Supplementary Table 10**), a pattern reflecting the higher ethylene production in SSL (TRTH2510) (**Figure 1C**). It is noticeable that another *ACO* ortholog (*ACO5*) was induced in LSL. The expression of transcripts involved in S-adenosylmethionine (SAM) synthesis and degradation, such as SAM synthases (*SAMS1*, *SAMS3*, LOC101247506, LOC101245012), and one SAM decarboxylase (*SAMDC2*; LOC101260400), were remarkably higher in LSL genotype, suggesting fluxes from ethylene biosynthesis towards polyamine synthesis. The up-regulation of polyamine pathway in LSL was further supported by the higher transcript levels of two arginine decarboxylases in chromosome chr1 and chr10. Concerning the other plant hormones, two homologs of the negative regulator of fruit ripening, involved in the oxidative degradation of abscisic acid (ABA), ABA 8’-hydroxylase (*CYP707A*) were up-regulated in LSL (TRTH1620) (*CYP707A2*, *CYP707A4*), and one (*CYP707A3*) in SSL (TRTH2510). Furthermore, a type 2C protein phosphatase (*PP2C*; LOC101257069) was induced in LSL, whereas different members of ABA *Pyl* receptors were also in either SSL (*PYL3*, *PYL9*) or LSL (*PYL9*). Regarding the transcripts related to indole-3-acetic acid (IAA), gibberellins, and jasmonate were mostly up-regulated in LSL, probably directing the fluxes towards to non-ethylene dependent metabolic pathways.

Several transcripts catalyzing metabolic steps of glycolysis and TCA cycle were up-regulated in both genotypes, including isoforms of glyceraldehyde-3-phosphate dehydrogenase, pyrophosphate-fructose 6-phosphate 1-phosphotransferase, fructose-bisphosphate aldolase, malate dehydrogenase, and aconitate hydratase (**Supplementary Table 10**). This finding suggests that these key routes of the primary metabolism were active regardless the shelf-life potential. Nonetheless, pyruvate decarboxylases (*PCD*s, LOC101256911, LOC101247173, LOC101246495), catalyzing the conversion of pyruvate to acetaldehyde with release of carbon dioxide as part of the fermentation process that generates ethanol, were significantly up-regulated only in LSL. This is in line with the higher ethanol content found in TRTH1620 (**Supplementary Table 3**).

Fatty acid metabolism was mostly enhanced in SSL compared to LSL, with 15 and 8 DEGs being up-regulated, respectively (**Supplementary Table 10**). Among the key up-regulated transcripts in SSL, there were 3 ketoacyl-CoA synthases, 3 oxoacyl-synthases, an acetyl-CoA carboxylase, and 2 acyl-coenzyme A oxidases, whereas in LSL, there were long chain acyl-CoA synthetases, phospholipases and stearoyl-[acyl-carrier-protein] 9-desaturases (**Supplementary Table 10**).

Transcripts participating in isoprenoid pathway proceeding through mevalonate (MVA) or methylerythritol phosphate (MEP) pathway were also found among DEGs (**Supplementary Table 10**). In particular, the expression of 1-D-deoxyxylulose 5-phosphate synthase (*DXS*), *DXR*, 3-hydroxy-3-methylglutaryl-coenzyme A reductases (*HMGR3*), 4-hydroxy-3-methylbut-2-en-1-yl diphosphate synthase (*HDS*), and *GGPS2* was higher in SSL, whereas other two orthologs of *HMGR* (*HMGR1*, *HMGR2*) in LSL. This interesting finding points to the up-regulation of isoprenoid pathway through MEP in SSL.

The behavior of carotenoids and apocarotenoids pathway-related aroma genes was also differentially affected in the two contrasting genotypes, as revealed by the identification of several key transcripts among DEGs (**Supplementary Table 10**). However, it was clear that this pathway was transcriptionally enhanced in SSL, that exhibited higher expression of *PSYs* (*PSY1*, *PSY2*), zeta-carotene desaturase (*zds*), 15-cis-zeta-carotene isomerase (*Z-ISO*) and nine-cis-epoxycarotenoid dioxygenase (*NCED1*). Only one transcript, β-carotene hydroxylase (*CrtR-b2*), was up-regulated in LSL, that might be associated to the higher lutein level determined in TRTH1620 (**Figure 3**), as well could potentially contribute to the metabolic flux towards the synthesis of ABA. No carotenoid cleavage dioxygenases (*CCD*s), with the aforementioned change in *NCED1* involved in ABA production, were differentially expressed between the two genotypes that could have explained the higher accumulation of β-damascenone in SSL.

Phenylpropanoid pathway, proceeding through shikimate, as well as the following pathways generating flavonoids, anthocyanins, lignins, were exclusively up-regulated in SSL (**Supplementary Table 10**). In particular, phenylalanine ammonia-lyase (*PAL*; LOC101243656), trans-cinnamate 4-monooxygenases (LOC101244196, LOC101244496) from the main phenylpropanoid pathway, as well as two *CHS*s (*CHS1*, *CHS2*), isoflavone reductase (*IFR*, LOC101265488), flavonol synthase/flavanone 3-hydroxylase (LOC101249699) and flavanone 3-dioxygenase (*F3’H*) showed higher expression in SSL than in LSL. A similar pattern was also found for anthocyanins, with several anthocyanidin 3-O-glucosyltransferases being up-regulated, as well for lignins, with caffeoylshikimate esterase (*CSE*) and shikimate O-hydroxycinnamoyltransferase (*HCT*) having higher transcript levels. Therefore, the stimulation of the aforementioned secondary metabolic pathways towards flavonoid and lignin generation, is clear in the case of SSL, at least at the transcript level.

Among the structural genes of the AsA pathways, we identified one AsA biosynthetic gene and two AsA recycling genes up-regulated only in SSL (**Supplementary Table 10**), which also showed higher AsA content than LSL. These were *GGP* in chr6, the key rate limiting step in AsA biosynthesis (Bulley and Laing, 2016; Fenech et al., 2021; Koukounaras et al., 2022), as well as monodehydroascorbate reductase (*MDHAR*) in chr2 (LOC101253550) and glutathione reductase (*GR*) from the AsA recycling pathway. By contrast, two ascorbate oxidase (*AO*) orthologs (LOC101252344, LOC101263571) and a nucleobase-ascorbate transporter (*NAT*; LOC101264718) were induced in LSL. With regard to B6 vitamin, the key gene of its biosynthesis, pyridoxal 5’-phosphate synthase (*PDX1.2*) was also up-regulated in LSL, which also exhibited higher accumulation levels (**Supplementary Table 6**). On the other hand, the fact that genes strictly related to oxidative stimuli, such as peroxidases (four orthologs), were up-regulated in LSL, probably suggesting its different cellular redox state, whereas transcript levels of catalase (*cat1*) were elevated in SSL (**Supplementary Table 10**).

One of the most profound findings of metabolomics analysis between TRTH2510 and TRTH1620 was that the selected LSL accumulated significantly more steroidal alkaloids and glycoalkaloids than SSL (**Supplementary Table 6**), although these differences were not relevant for the other LSL and SSL genotypes (**Table 1**). Transcriptome analysis in fruits of the two cultivars revealed that these remarkable differences may be attributed to the up-regulation of four genes in LSL, including 11-β-hydroxysteroid dehydrogenase (LOC101264635), 3-β-hydroxysteroid-Delta(8), Delta(7)-isomerase (LOC101259646), squalene monooxygenase (LOC101250012), and a 2-oxoglutarate-dependent dioxygenase (LOC101246594) (**Supplementary Table 10**). The latter gene corresponds to *GAME31* (Solyc02g062460) that catalyzes the hydroxylation of the bitter flavor α-tomatine in tomato fruit, the first committed step toward esculeoside A (Cárdenas et al., 2016).

In line with fatty acid metabolism, that was more stimulated in SSL, genes related to lipid-derived VOCs produced through acetyl-CoA, were also up-regulated mostly in SSL. In particular, linoleate 9S-lipoxygenase (*TomloxB*) and *TomloxC* were more expressed in TRTH2510 (**Supplementary Table 10**), with the latter being crucial in C5 and C6 lipid-derived volatiles biosynthesis. Another lipoxygenase, linoleate 13S-lipoxygenase (*TomloxD*) was up-regulated in TRTH1620. Regarding benzenoids- and nitrogenous aroma-related VOCs, three genes were up-regulated in SSL and suppressed in LSL, including an UDP-glycosyltransferase 74F2 (LOC101247047) and two flavin-containing monooxygenases (*FMO*s; LOC101254042, LOC101254343).

**Figure 5:**
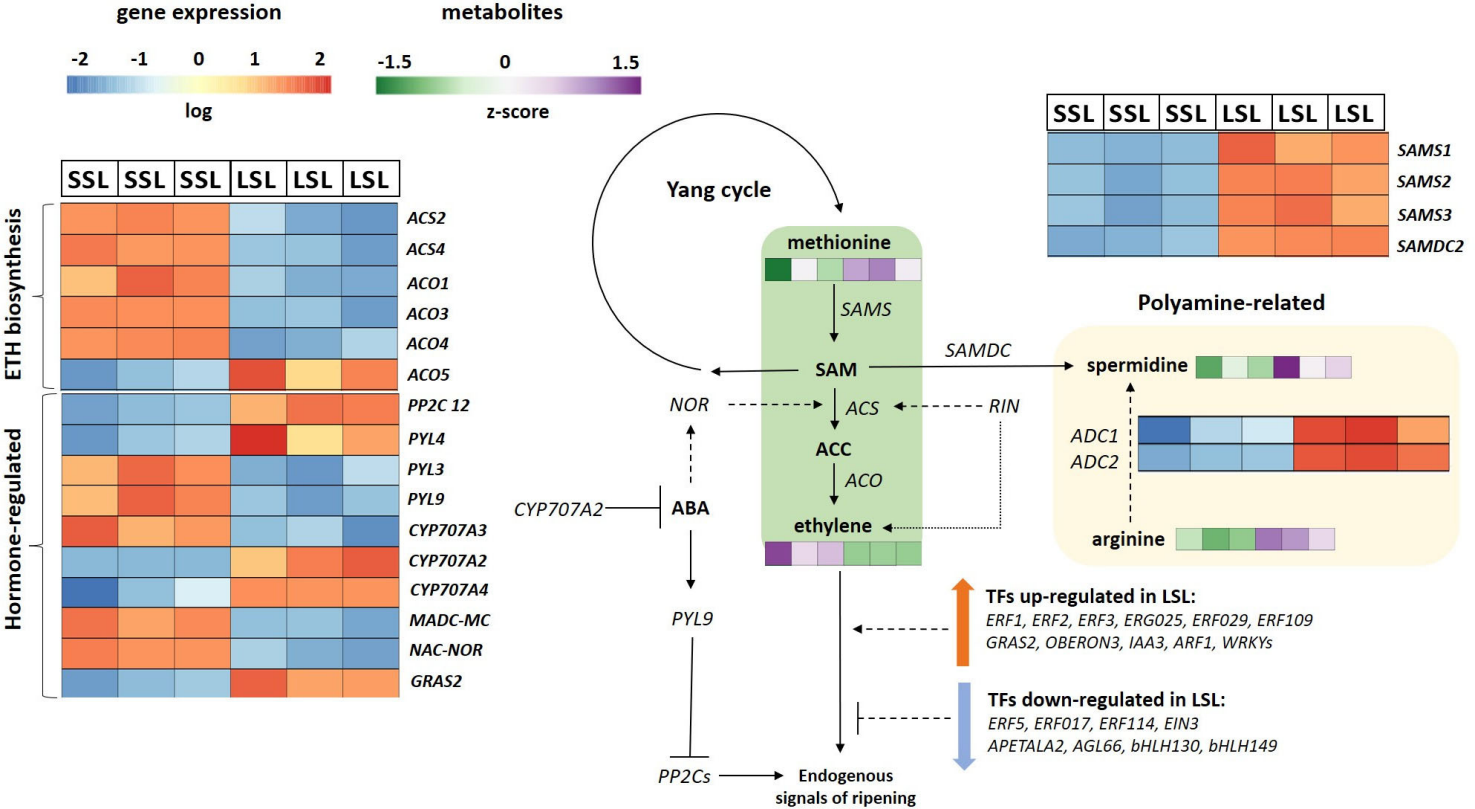
Ethylene related pathways in the SSL (TRTH2510) and LSL (TRTH1620) genotypes. The expression levels of DEGs are reported in boxes with a blue-to-red scale, while the accumulation of DAMs is in boxes green-to-purple scale. For each transcript/metabolite, the expression/abundance levels are represented by heatmaps. ABA, abscisic acid; ACC, 1-aminocyclopropane-1-carboxylate; *ACS*, ACC synthase; *ACO*, ACC oxidase; *ADC*, arginine decarboxylase; *ARF*, auxin response factor; *CYP707A*, ABA 8’-hydroxylase; *EIN3*, ethylene insensitive 3; *ERF*, ethylene-responsive transcription factor; *NOR*, non-ripening; *PP2C*, type 2C protein phosphatase; *PYL*, ABA PYL receptors; *RIN*, ripening-inhibitor; SAM, S-adenosylmethionine; *SAMS*, SAM synthase; *SAMDC*, SAM decarboxylase.

Several transcription factors (TFs) were identified as DEGs between TRTH1620 and TRTH2510 (**Figure 5**; **Supplementary Table 10**). Among them, a broad number of genes related to fruit development and ripening were up-regulated in SSL and suppressed in LSL, including MADS-box TFs (*MADS-MC*) or *NAC-NOR* that regulates ethylene production, carotenoid accumulation and fruit firmness (Li et al., 2018b; Gao et al., 2020), or the floral homeotic *AGAMOUS* protein (*TAG1*), the agamous-like MADS-box protein *AGL66* and an *APETALA2*-like protein. Of particular interest is the fact that the *GRAS2* protein, a negative regulator of fruit ripening (Li et al., 2018a), was significantly induced in LSL and repressed in SSL. A broad number of ethylene-responsive TFs (*ERF*s) were also up-regulated in both genotypes, regardless of postharvest potential. In particular, *ERF1*, *ERF2*, *ERF3*, *ERF025*, *ERF027*, *ERF109* and *ERFD2* were up-regulated in LSL, whereas, *ERF5*, *ERF017*, *ERF114*, and *EIN3* were up-regulated in SSL. The same trend was evident for the large TF families of *bZIP* and *MYBS*, with different gene orthologs being induced in either accession (**Supplementary Table 10**). By contrast, TFs of the wide WRKY family (*WRKY17*, *22*, *23*, *40*, *57*, *70*), as well as IAA-responsive factors (*OBERON3*, *IAA3*, *auxin response factor 1*), were exclusively induced in LSL, whereas *MYC* TFs (*MYC1*, *MYC2*) and *bHLH* TFs (*bHLH130*, *bHLH149*) were induced in SSL.

## 4 Discussion

### 4.1 Ethylene and ABA mediate transcriptome reprogramming related to improved shelf-life

Tomato ripening is a complex and dynamic process characterized by the coordinated transcriptional regulation leading to metabolic remodeling of key compounds related to fruit quality, nutritional value, taste and aroma. Over the last decades, RNA-seq approaches have been widely employed as an efficient tool to identify genes involved in metabolic pathways contributing to fruit quality (D’Esposito et al., 2017; Sacco et al., 2019) and aroma (Peng et al., 2022; Villano et al., 2023). The vast majority of the transcriptional responses during ripening are genotype-specific, highlighting the molecular events leading to metabolic clues that are biologically interesting and important from a horticultural perspective. However, modern cultivated tomatoes have narrow genetic diversity limiting their improvement potential. Due to the long-lasting emphasis of breeders on yield and postharvest potential, flavor-associated VOCs have been ignored to a great extent (Gao et al., 2019). Herein, we present a comprehensive survey of metabolome profile of a collection of tomato germplasm, consisting of underutilized traditional cultivars, differing in postharvest potential (short or long). In combination with the transcriptome profile of the best SSL *versus* the best LSL varieties, hub genes explaining the observed differences among them were also pinpointed, setting new breeding targets to develop more nutritious and flavorful tomato varieties, with enhanced shelf-life.

During tomato climacteric ripening, a burst in ethylene production occurs through the activation of an autocatalytic biosynthesis circuit, with both ethylene signaling and transcriptional regulation possessing pivotal roles in this process (Wang et al., 2020). Our 2-year experiments confirmed the classification of Greek landraces as SSL or LSL, suggesting that this trait is not under strong environmental control. Despite the fact that SSLs mostly exhibited higher ethylene production than LSLs, correlating with their poor shelf-life performance, there were some accessions like TRTH1460, TRTH0500, TRTH0550, characterized by high ethylene production rates. Although their transcriptome was not analyzed herein, this bizarre finding merits further attention to investigate what makes these accessions not overripe during postharvest, especially taking in consideration that ethylene is the end-product of the pathway.

A proposed model for the ethylene-dependent pathways differentially regulated between the two genotypes is presented in **Figure 5**. The increased ethylene production in SSL seems to be orchestrated by the transcription of the key ethylene biosynthetic genes (*ACS2*, *ACS4*, *ACO1*) responsible for pre- and post-climacteric ethylene biosynthesis (Van de Poel et al., 2012). *MADS-RIN*, is a crucial early regulator of fruit ripening (Vrebalov et al., 2002) binding to the promoters of *ACS2* and *ACS4*, with hundreds of gene targets controlling changes in color, flavor, texture, and taste during tomato fruit ripening. Its expression was nearly 2-fold lower in LSL compared to SSL (**Supplementary Table 8**), correlating with the lower ethylene production of this genotype, and probably with other important ripening-induced processes. Additionally, the *MADS-MACROCALYX* (*MADS-MC*) belonging to the *APETALA1/FRUIT-FULL* (*AP1*/*FUL*) subfamily and being part of the RIN-MC fusion protein, was also down-regulated in LSL. Down-regulation of the *RIN-MC* fusion gene has been found to accelerate the yellow ripening in *rin* mutants (Li et al., 2018b). Furthermore, it has been previously reported that the RIN-MC fusion protein is an active TF with a repressor function controlling fruit softening and color, through *PG*s, *PME*s, *PSY*s and *GGPPS2* (Li et al., 2018b; Li et al., 2020). Our findings confirmed the earlier reports, except for *PSY*s, which were down-regulated in SSL, suggesting that the exact function of this TF remains to be fully elucidated. Although *MADS-MC* alone is not considered as a master regulator of ripening (Wang et al., 2020), its down-regulation in LSL, along with the lower transcript levels of *RIN*, reveal interesting aspects of interaction with the other TFs contributing to the improved postharvest potential of TRTH1620, that merits further exploitation. Consistently, the expression of *NAC-NOR* that also plays a significant role in fruit ripening, was also up-regulated in SSL. The NAC-NOR truncated protein binds to the promoters of *ACS2*, *GGPPS2* and *PL*, stimulating ethylene biosynthesis, carotenoid accumulation and fruit softening, respectively (Gao et al., 2020). An induction of the transcript levels of these genes was also evident in this study. Several studies have shown that members of the *GRAS* TFs are widely considered as negative regulators of ripening through controlling different metabolic processes i.e. *GRAS38* (lycopene content and ethylene production) or *GRAS4* (ethylene production through binding to the promoters of *ACO*) (Liu et al., 2021). In our study, we identified another member of this family, *GRAS2*, which was significantly down-regulated in SSL. Previously, down-regulation of this TF was found to suppress the expression of down-stream genes related to fruit development, such as gibberellin (GA) biosynthesis and signal transduction pathways (Li et al., 2018a). Indeed, members of both pathways (*GA2ox2* from GA biosynthesis, and *20ox-3* from GA inactivation) were repressed in SSL, which showed significantly lower *GRAS2* transcript levels. In the same study, silencing *GRAS2* has also caused a decrease in cell size and cell expansion through regulating XTHs. However, this observation does not hold true in this study, as the up-regulation of *GRAS2* in LSL was accompanied by higher transcript levels of *XTH*s and xyloglucan galactosyltransferases (*XLT*s). This finding suggests that *GRAS2* regulation mode is more complicated and probably involves other TFs.

Apart from ethylene, ABA also functions as a significant hormone in the control of climacteric fruit ripening by triggering the expression of many ethylene-independent genes (Mou et al., 2015). The core ABA signaling components consist of *Pyl* receptors, *PP2C*s, and sucrose nonfermenting-related protein kinases. In total, 15 members of *PYL*s have been characterized in tomato, having distinct properties (González-Guzmán et al., 2014). Herein, *PYL3* and *PYL9* were induced in SSL, whereas *PYL4* in LSL. Among them, *PYL4* has been linked to drought tolerance (Li et al., 2022b), while *PYL3* has not been characterized yet. Interestingly, silencing of *PYL9* was found to retard ripening, increase mesocarp thickness, resulting to conical/oblong and gourd-shaped fruits (Kai et al., 2019). This is presumably related to the longer shelf-life of TRTH1620, which showed lower *PYL9* transcript levels than TRTH2510, and oblong fruit shape. On the other hand, *PP2C*s are generally known to act negatively in ABA signaling regulation with additive functions. For instance, *PP2C1* retards fruit ripening, through blocking ABA and ethylene accumulation (Zhang et al., 2018), whereas *PP2C3* plays an important role in the regulation of fruit ripening and fruit glossiness in tomato (Liang et al., 2021). On the basis of these considerations, another member of this family, *PP2C12*, was induced in LSL, probably correlating with the longer storability of this genotype, although its functional role has not been determined yet. Another group of ABA regulators are the *CYP707A* genes encoding ABA 8′-hydroxylases, which catalyze the oxidative catabolism of ABA. Although different isoforms seem to have distinguished roles, it was of particular interest that the expression of *CYP707A2* was dramatically up-regulated in LSL (log2FC > 4) (**Figure 5**; **Supplementary Table 10**), suggesting that it contributes to the catabolism of ABA in fruit. Previously, silencing of *CYP707A2* significantly changed the transcripts of a broad number of ABA-responsive and ripening-related genes, including those related to lycopene-synthesis or cell wall-degrading genes (Ji et al., 2014). The key gene in ABA biosynthesis, *NCED*, was also decreased in LSL, but its role will be discussed below.

Polyamines are aliphatic amines present in all living organisms with intriguing functions in fruit development and ripening (Gao et al., 2021). In plants, polyamines and ethylene act competitively as double-edged swords in ripening and senescence processes (Mehta et al., 2002). At the same time, exogenous application of polyamines can enhance postharvest life and quality of fruits (Zahedi et al., 2019). On the basis of these considerations, LSL, the genotype producing low ethylene levels, accumulated more methionine, and had higher transcript levels of *SAMS* and *SAMDC* (**Figure 5**). It was therefore demonstrated that, in genotypes with long postharvest potential, the flux from Yang cycle is preferably directed towards polyamine synthesis, fueling spermidine accumulation, and not towards the ethylene synthesis. The up-regulation of polyamine pathway in LSL was further supported by the higher arginine levels, accompanied by higher transcript levels of two arginine decarboxylases genes (*ADC*s), but not of the other key genes of the pathway, i.e. ornithine decarboxylase, spermidine synthase, spermine synthases, suggesting that polyamine biosynthesis is regulated at both transcriptional and translational level during ripening. In line with our findings, overexpression of *ADC* in tomato resulted to elevated levels of polyamines accompanied by enhanced shelf-life due to the decrease in ethylene levels (Gupta et al., 2019), confirming the link between high ADC transcript levels, low ethylene accumulation and improved postharvest potential.

### 4.2 Isoprenoid-related genes are valuable candidates to unravel distinctive quality and postharvest properties

**Figure 6:**
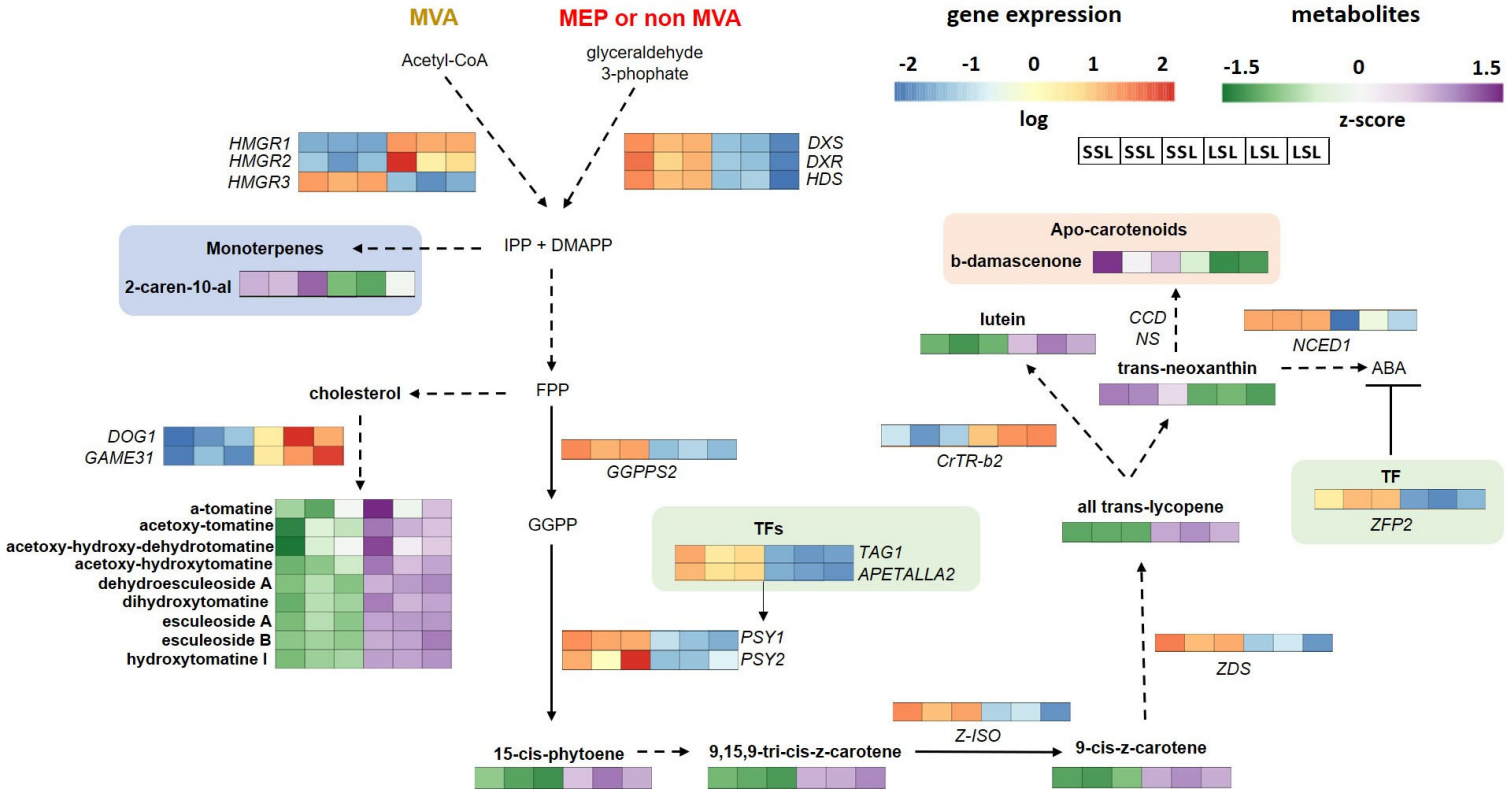
Isoprenoid, carotenoid and related biosynthetic pathways, in the cytosol (mevalonate, MVA) and in plastids (methyl-D-erythritol phosphate, MEP), of SSL (TRTH2510) and LSL (TRTH1620) genotypes, producing monoterprene-related VOCs, apocarotenoids, and steroidal alkaloids and glycoalkaloids. Only significant DEGs and DAMs are presented in the pathways*. CrtR-b2*, beta-carotene hydroxylase; CCDs, Carotenoid cleavage dioxygenase; DMAPP, dimethylallyl diphosphate; *DOG1*, DELAY OF GERMINATION 1; *DXR*, -deoxy-D-xylulose-5-phosphate reductoisomerase; *DXS*, 1-Deoxy-D-xylulose 5-phosphate synthase; FPP, farnesyl diphosphate; GGPP, geranylgeranyl pyrophosphate; *GGPPS*, geranylgeranyl pyrophosphate synthase; *HDS*, 4-hydroxy-3-methylbut-2-en-1-yl diphosphate synthase; *HMGR*, 3-hydroxy-3-methylglutaryl-coenzyme A reductase; IPP, isopentenyl diphosphate; *NCED*, nine-cis-epoxycarotenoid dioxygenase; *PSY*, phytoene synthase; *TAG1*, floral homeotic protein AGAMOUS; *ZDS*, zeta-carotene desaturase; *ZFP2*, zinc finger transcription factor; *Z-ISO*, 15-cis-zeta-carotene isomerase; NS, non-significantly different.

Through the isoprenoid-related pathways, various volatile compounds, such as monoterpenoids (C10) and sesquiterpenoids (C15) found mostly in unripe fruits, as well as carotenoids, whose synthesis, increased in ripe fruits, can be produced (Rambla et al., 2014). These compounds are synthesized from isopentenyl diphosphate (IPP) and its isomer dimethylallyl diphosphate (DMAPP), either through the cytosolic MVA or the plastid MEP pathway. Herein, the terpenoid accumulation and the transcript levels of the underlying pathway genes revealed differences between LSL and SSL, with MEP pathway being clearly stimulated in SSL, and MVA pathway more activated in LSL (**Figure 6**). Previously, the early transcriptional stimulation of MVA pathway that produces the building blocks for terpenoids, was correlated with reduced susceptibility of apples during postharvest storage (Roberto et al., 2022). Key up-regulated genes in the case of SSL included *DXS*, *DXR*, and *HDS* of MEP pathway, whereas in the case of LSL, two out of the three *HMGR* transcripts (MVA pathway). The accumulation of the monoterpene, 2-caren-10-al, was also enhanced in SSL, but this compound does not seem to contribute to tomato aroma. Within tomato pangenome that explored wild promoter regions, *DXR* is the only candidate gene showing a wild allele for its promoter region, whereas the cultivars possessing this wild allele have higher *DXR* expression compared to those carrying the common allele (Gao et al., 2019). This observation reinforces the hypothesis that modern breeding targets should be further explored by utilizing underutilized tomato germplasm to develop new lines with superior attributes.

Carotenoid biosynthesis takes place in plastids, starting with *PSY*, the rate-limiting enzyme, catalyzing the first committed step of the pathway by condensation of two molecules of GGPP to produce the C40 hydrocarbon 15-cis-phytoene (Zhou et al., 2022). Two are the main *PSY* genes found within tomato genomes, being expressed during fruit ripening. Although both of them were up-regulated in SSL, this increase in expression levels did not lead to high accumulation levels of 15-cis-phytoene or ζ-carotene (**Figure 6**). Silencing *PSY1* has been previously related to the yellow-flesh tomato phenotype (Kang et al., 2014). Up-next, *Z-ISO* and *ZDS* are responsible for the conversion of phytoene to lycopene (Ampomah-Dwamena et al., 2019). Again, although transcript levels of both genes were remarkably stimulated in SSL, the accumulation of ζ-carotene and lycopene was higher in LSL, suggesting either a feedback regulation, or a post-transcriptional regulation of the pathway. As carotenoid accumulation is tightly linked to other ripening-related signals such as ethylene biosynthesis and perception, there may be other regulators far upstream this pathway. Previously, the re-direction of fluxes from the ethylene biosynthesis towards polyamine synthesis via overexpression of a yeast *SAMDC* in tomato has led to enhanced accumulation of lycopene, accompanied by improved vine-life (Mehta et al., 2002). This regulatory mechanism through polyamines may be relevant in the case of LSL. Additionally, the MADS-box ripening regulators including Tomato *AGAMOUS-LIKE1* (*TAGL1*), *FRUITFULL1* (*FUL1*), and *FUL2*, can positively control the expression of carotenoid biosynthetic genes, and negatively the expression of genes of the downstream reactions, through binding to their promoters (Stanley and Yuan, 2019). Indeed, *TAGL1* was induced in SSL, probably leading to the induction of *PSY*s, *Z-ISO* and *ZDS*, but *FUL*s were not differentially expressed between genotypes. This is in line with another study, indicating that silencing of these genes did not alter the expression of *PSY1* in tomato (Bemer et al., 2012), questioning its role in regulating carotenoid biosynthesis. Similarly, TFs involved in hormone synthesis and signaling, such as an *APETALA2/ERF* protein, can also promote the expression of carotenoid biosynthetic genes (Karlova et al., 2011), as reported herein too.

As carotenoids are precursors of ABA, which also serves as an important trigger of fruit ripening along with ethylene, complex interactions between upstream and downstream reactions in the carotenoid pathway are rational (Mou et al., 2015). Therefore, a positive correlation between ABA and carotenoids have been widely reported (Tung et al., 2008; Mou et al., 2015). Different members of *NCED* family are up-regulated during tomato ripening (Ilahy et al., 2019), participating in the catabolism of carotenoids towards ABA generation (Sun et al., 2012), thus being important determinants of fruit coloration. Although ABA was not measured in this study, the up-regulation of *NCED1* in SSL (**Figure 6**) probably indicated its higher accumulation compared to LSL. However, previous reports (Diretto et al., 2020) have also shown an increased shelf-life in tomato fruits following over-expression of genes involved in carotenoid late pathway (e.g. *lycopene β-cyclase*, *LCYb*), with a simultaneous increase in total carotenoids and ABA, thus proving the existence of a complex and still not fully elucidated network of interactions within the aforementioned components and ethylene. Among the four *NCED* and the four *CYP707A* genes in the ABA metabolism pathway, *NCED1* and *CYP707A2*, respectively, are the most important in orchestrating ABA levels within fruit (Kai et al., 2019). Interestingly, *NCED1* (ABA biosynthesis) was up in SSL, while *CYP707A2* (ABA catabolism) (**Figure 5**) was down in SSL, further pointing to the over-accumulation of ABA in TRTH2510 (SSL). On the other hand, the zinc finger transcription factor *ZFP2* has been reported to block fruit ripening by negatively regulating ABA biosynthesis, with *ZFP2*-overexpressing containing more lycopene compared to WT (Weng et al., 2015). Its expression was stimulated in SSL, but once again, it did not correlate with carotenoid content, suggesting a different target than the carotenoid biosynthetic path.

A key group of tomato VOCs derived from the degradation of carotenoids, thus directly linked to their accumulation, is referred as apocarotenoids, that are important contributors of tomato ripe fruit aroma conferring a typical floral/fruity odor (Klee and Tieman, 2018). Most of them are directly produced by the oxidative cleavage catalyzed by carotenoid cleavage dioxygenases (CCDs), except for β-damascenone, which is highly abundant in ripe fruits (Wang et al., 2016). Previously, apocarotenoid-deficient fruits are also significantly less preferred by consumers, probably due to their correlation with sweetness as in the case of geranial (Tieman et al., 2012). Another important apocarotenoid, the fruity-flavor β-ionone, is produced via the oxidative breakdown of β-carotene, which predominates orange-fruited genotypes, whilst geranial, neral, and citral, providing the lemon-note, mostly contributes as a background aroma odor (Distefano et al., 2022). Herein however, the only apocarotenoid compound which was found among DAMs over-accumulated in SSL was β-damascenone (**Figure 6**), which provides an odor of apple, rose, and honey to tomato fruit (Martina et al., 2021). Although this compound was long regarded as important for tomato aroma, mostly because of its extremely low reported odor threshold (Vogel et al., 2010), latter studies suggest that it can no longer be considered as a high-priority target for flavor enhancement as it is not associated with the intensity of tomato flavor (Tieman et al., 2012; Gao et al., 2019). Interestingly, *TomLoxC*, which is deemed to be crucial in C5 and C6 lipid-derived volatiles biosynthesis and apocarotenoid production (Chen et al., 2004), was also up-regulated in SSL. Its rare allele has been generally recovered in modern elite breeding lines, as *TomLoxC* is regarded as the key gene in flavor-associated lipid- and carotenoid-derived VOCs (Gao et al., 2019).

### 4.3 *GAME31* and *DOG1* are key regulators of alkaloid accumulation in ripe tomatoes

Steroidal alkaloids and steroidal glycoalkaloids are N-containing antinutritional compounds, predominantly found during the early stages of tomato fruit ripening, with α-tomatine and dehydrotomatine being mainly accumulated in green fruits (Itkin et al., 2011). Galactosyltransferase (*GAME*) genes including *GAME1*, *GAME5*, *GAME9*, *GAME17*, *GAME18*, *GAME31* have been previously reported to participate in alkaloid formation in tomato fruit, responsible for the conversion of cholesterol and tomatidine to non-bitter molecules, such as esculeoside A (Itkin et al., 2013; Cárdenas et al., 2016; Szymański et al., 2020). Among these *GAME*s, we identified a dioxygenase dependent on 2-oxoglutarate *GAME31*, which was remarkably up-regulated in LSL, and associated with enhanced levels of these alkaloid compounds (**Figure 6**). According to previous studies, the gene performs the conversion of α-tomatine to hydroxytomatine and has been highly conserved through domestication to ensure a less bitter and harmless tomato fruit (Cárdenas et al., 2016). Interestingly, another TF of the *bZIP* family, the *DELAY OF GERMINATION 1* (*DOG1*) that has been recently identified as a principal regulator of the pathway targeting *GAME*s transcription (Zhao et al., 2023), was also enhanced in LSL, suggesting that the pathway is activated.

### 4.4 *GGP*, *MDHAR* and *GR* are key determinants of AsA levels

**Figure 7:**
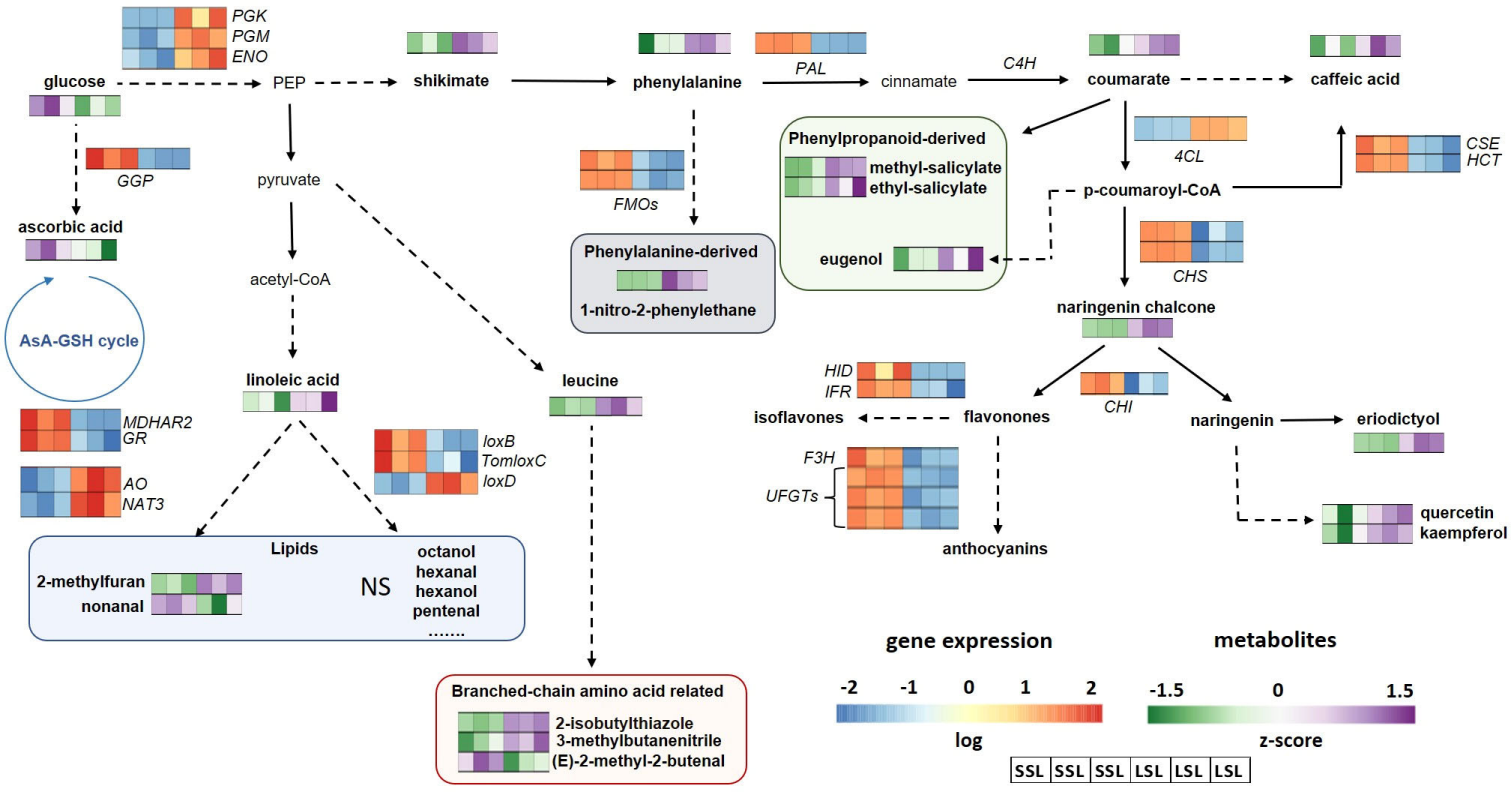
Metabolic pathways related to flavonoids, VOCs and AsA in the SSL (TRTH2510) and LSL (TRTH1620) genotypes. Only significant DEGs and DAMs are presented in the pathways. *AO*, ascorbate oxidase; AsA, ascorbic acid; *C4H*, cinnamate-4-hydroxylase; *4CL*, 4-coumarate-CoA ligase; *CHI*, chalcone-flavonone isomerase; *CHS*, chalcone synthase; *CSE*, caffeoylshikimate esterase; *ENO*, enolase; *F3H*, flavanone 3-dioxygenase; *FMO*, flavin-containing monooxygenase; *GGP*, GDP-L-galactose phosphorylase; GSH, glutathione; *GR*, glutathione reductase; *HCT*, shikimate O-hydroxycinnamoyltransferase; *HID*, hydroxyisoflavanone dehydratase; *IFR*, isoflavone reductase; *lox*, lipoxygenase; *MDHAR*, monodehydroascorbate reductase; *NAT*, nucleobase-ascorbate transporter; *PAL*, phenylalanine ammonia-lyase; *PGK*, phosphoglycerate kinase; *PGM*, phosphoglycerate mutase; *UFGT*, flavonoid 3-O-glycosyltransferase; NS, non-significantly different.

Ascorbate (AsA) is one of the most abundant and powerful antioxidants present in plant tissues and in particular in tomato fruit (Mellidou and Kanellis, 2017). It has been well established that AsA is predominantly accumulated through the so-called _L_-galactose pathway, with GDP-_L_-galactose phosphorylase (GGP) that catalyzes the first committed step of the pathway, serving as the rate limiting step (Bulley et al., 2012; Mellidou et al., 2012; Koukounaras et al., 2022). Indeed, in our study, AsA levels correlated with *GGP* transcript levels, with SSL being also richer in this nutritious molecule than LSL (**Figure 7**). The accumulation of AsA within tomato fruit is also dependent on AsA recycling, with *MDHAR*, *DHAR* and *GR* serving as key genes in regenerating its reduced form (Noctor et al., 2016). In line with the current consensus on AsA recycling pathway, the high AsA levels determined in SSL can be attributed to the enhanced transcript levels of *MDHAR2* and *GR*. The expression of *MDHAR* has been previously related to total AsA content during tomato ripening (Mellidou et al., 2012). Additionally, AsA oxidation through an ortholog of ascorbate oxidase (*AO*) was stimulated in LSL, suggesting its involvement in cell growth and expansion to support fruit growth, as previously reported in melon (Chatzopoulou et al., 2020). Therefore, the AsA enrichment of SSL can be attributed to the enhanced expression of key AsA biosynthesis, recycling and oxidation determinants. Furthermore, in tomato, there are at least 12 nucleobase–ascorbate transporters (*NAT*s) that participate in the long-distance phloem-mediated transport of AsA. In ripe fruits of LSL, *NAT3* was significantly induced. This specific gene ortholog has been found to be widely distributed and expressed in all tomato organs, with its expression values being higher at 10 and 20 days after pollination (Cai et al., 2014), suggesting its putative role earlier in fruit development, that cannot efficiently explain AsA-related differences between LSL and SSL. None of the regulatory TFs able to “unlock” the AsA synthesis either at the transcriptional or the post-translational level (Mellidou and Kanellis, 2023) were differentially expressed between the two genotypes, suggesting that the observed differences were mostly due to the transcription of structural pathway genes.

### 4.5 Discrepancies in the phenylpropanoid pathway between transcript and metabolite level

Tomato fruit is a good source of polyphenolic compounds such as naringenin chalcone, rutin, quercetin, hydroxybenzoic acid, caffeic acid, naringenin and flavonones (Lu and Zhu, 2022) that contribute to tomato fruit color and volatile production during ripening (Tieman et al., 2006). As shown in **Figure 7**, the phenylpropanoid pathway proceeding through phenylalanine provides the precursors for the production of flavonoids and lignin, with *PAL* being the first rate-limiting step of the pathway, redirecting the flux from the primary metabolism to the synthesis of polyphenolic compounds. In the following steps, cinnamate is converted to coumarate and coumarate-CoA in reactions catalyzed by *C4H* and *4CL*, respectively. Surprisingly, KEGG enrichment analysis revealed an up-regulation of *PAL* and *4CH* transcripts in SSL, while both phenylalanine, and coumarate were most accumulated in LSL, an observation suggesting a feedback regulation of the pathway. In fact, the breakdown of glucose to PEP, shikimate and phenylalanine was also stimulated in LSL at both metabolite and transcript level (induction of *PGK*, *PGM*, *ENO*). The only gene that was positively correlated with the high phenylpropanoid levels recorded in LSL was 4-coumarate-CoA ligase (*4CL*). This gene is responsible for the esterification of coumarate to coumaroyl-CoA, that undergoes a large number of downstream reactions to generate the majority of phenylpropanoid compounds (Alberstein et al., 2012). The expression of this gene was also positively correlated with the accumulation of caffeic acid and coumarate, as well as with enhanced skin resistance and hardness in the fruits of LSL. This is in disagreement with previous studies in tomato concerning another *4CL* gene copy in chr6 (Solyc06g035960), suggesting a complex network of the different gene orthologs. Two other genes related to lignin biosynthesis caffeoylshikimate esterase (*CSE*) and shikimate O-hydroxycinnamoyltransferase (*HCT*) were also up-regulated in SSL, suggesting an up-regulation of the pathway, although no lignin was quantified in this study. High levels of lignins adversely influence consumer preferences for tomato, thereby it is an undesirable trait for the fresh market (Lu and Zhu, 2022).

The specific flavonoid pathway starts with chalcone synthase (*CHS*), which converts coumaroyl-CoA to naringenin chalcone. This reaction is further prone to isomerization, hydroxylation and oxidation to generate the primary flavonoids (Ilahy et al., 2019). Overall, mature fruits contain low level of flavonoids and even lower or zero levels of anthocyanins. Again, the two CHSs orthologs were induced in SSL, but naringenin chalcone was low in this genotype compared to LSL. Chalcone isomerase is the next limiting step in flavonoid biosynthesis that isomerize naringenin chalcone to naringenin. Similar to previous reports, *CHI* was negatively correlated with naringenin chalcone (Yang et al., 2022). The following steps proceeding towards flavonones and anthocyanins were up-regulated in SSL (at the transcript level), whereas the steps towards quercetin, kaempferol, and eriodictyol were induced in LSL (at the metabolite level), respectively, suggesting the different pathway reprogramming in the two genotypes. Rutin is considered as the major glycosylated flavonoid in ripe fruits, followed by phloretin (Ilahy et al., 2019). This observation is also confirmed herein, although no remarkable differences were identified between SSL and LSL genotypes (**Supplementary Table 6**).

Overall, these discrepancies between transcripts and metabolites could be due to the feedback inhibition of the flavonoid pathway, upstream the chalcone synthase and isomerase steps, as previously reported in tomato (Verhoeyen et al., 2002; Alberstein et al., 2012). Therefore, it can be hypothesized that the enhanced transcription of both *CHS* and *CHI* in SSL utilizes coumaroyl-CoA and naringenin chalcone, and that the depletion of this pool in SSL removes a point of negative feedback in flavonoid synthesis, leading to the induction of regulatory genes of the pathway. Another possible explanation is that since the entire fruit was used for both metabolomics and transcriptomics, local tissue-specific different (pericarp, septa, columella, placenta, locular gel) were not taken into consideration. This tissue-oriented differentiation holds true in the case of ethylene biosynthetic pathway during tomato fruit ripening (Van de Poel et al., 2014) that could have also affected the regulation of phenylpropanoids herein in a tissue-specific manner. In this case, the observed inconsistencies could be recovered using single-cell transcriptomics, as the bulk RNA-sequencing of a given tissue may camouflage small changes at the cell level, especially in pathways such as phenylpropanoids that produce diverse compounds with dissimilar functions. Through single-cell technologies, it will be possible to build a high-resolution expression atlas of cells that could be a key resource (Jovic et al., 2022), especially in fruit tissues that undergo complex and uneven metabolic processes in a tissue-specific way.

### 4.6 Volatiles accumulation is dependent on genotype and relates to ethylene production

Fatty acids such as the polyunsaturated linoleic (c18:2) are the main precursors of fruit aroma volatiles, also known as green leaf volatiles, being synthesized either via β-oxidation or by means of lipoxygenases (LOX). Linoleic acid was remarkably higher in LSL, suggesting a stimulated flux towards the synthesis of C18 fatty acids. Among the five *LOX* genes expressed in tomato during ripening, using linoleic and linolenic acids as substrates, *TomloxC* was significantly down-regulated in LSL. This gene is widely deemed to be essential not only for the generation of fruit C6 volatiles (Chen et al., 2004), but also for synthesis of C5 volatiles, including 1-penten-3-one, (*E*)-2-pentenal, 3-pentanone, 1-pentanol, and 1-penten-3-ol (Zhu et al., 2019). Indeed, silencing *TomloxC* in tomato resulted to a 75% reduction in the levels of several C5 flavor volatiles, including pentanal (Shen et al., 2014). However, in melon, pentanal biosynthesis has been clearly related to *PCD* transcript levels, suggesting a link between fermentation and aroma production (Wang et al., 2019). Several *PCD*s were up-regulated in LSL, in line with ethanol and pentanal production (although no statistical differences in the second case), suggesting some interesting links between fermentation and volatile production. Another *LOX* gene, *TomloxD* also generating 13-hydroperoxides was up-regulated in LSL, whereas *TomloxB*, which was up-regulated in SSL, but this gene is not involved in the synthesis of C6 volatile compounds in the fruit. With regard to metabolites, the only statistically significant induced in LSL was 2-methylfuran, providing a chocolate odor (Li et al., 2019). Furthermore, (*E*)-2-heptenal, 1-hexanol, and hexanal, which are major contributors to the green/grassy aroma (Villano et al., 2023), were mostly accumulated in LSL, although the differences between genotypes were not statistically significant due to the great variability among replicates (**Supplementary Table 3**). On the other hand, nonanal levels, contributing to the pleasant aroma notes of ripe fruits, were higher in SSL (Ilahy et al., 2019). Additionally, (*E*)-2-hexenal (green, leafy), (*Z*)-3-hexenal (green, leafy), and 2-ethylfuran (coffee and chocolate) were also up-regulated in SSL, yet not statistically different. These results reveal interesting aspects of lipid-derived VOCs that were differentially accumulated in contrasting genotypes.

Branched-chain amino acid (BCAA) derived compounds are also major contributors to tomato aroma, including 3-methylbutanal (musty, peach), 2-methylbutanal (musty, coffee), 3-methylbutanol (musty, earthy), and 2-isobuthylthiazole (green, earthy) (Bizzio et al., 2022; Distefano et al., 2022). The majority of these compounds are derived through the reversible conversion of leucine/isoleucine into α-ketoacids, catalyzed by BCAA transferases (*BCAT*s). A chloroplastic *BCAT2* gene copy mapped in *chr12* (LOC101253875) was induced in SSL (**Supplementary Table 10**), whereas leucine accumulation was repressed (**Figure 7**). This finding implies this gene ortholog probably participates in BCAA synthesis rather than BCAA catabolism, as it is located in the chloroplast and not in mitochondria (Maloney et al., 2010). However, as there are different *BCAT*s members within the tomato genome that can mediate both synthetic and catabolic reactions of BCAA-derived VOCs, more functional characterization is required to explore the combined action of various classes of these enzymes. On the other hand, in LSL, the accumulation of both leucine as well as the nitrogen- and sulfur-containing compounds 2-isobuthylthiazole (wasabi, leafy odor) and 3-methylbutanenitrile (pungent) were induced, suggesting that the pathway is stimulated towards the production of these compounds. These leucine-derived compounds are very abundant in tomato fruit, being synthesized by the conjugation of 3-methylbutanal with cysteine (Liscombe et al., 2022; Kaur et al., 2023). However, the underlying key gene, tetrahydrothiazolidine N-hydroxylase (Wang et al., 2023), was not differentially expressed between genotypes, suggesting that other factors may also be important. All the above-mentioned BCAA-derived compounds, found in either of the two genotypes, have been associated with liking by consumers (Kaur et al., 2023).

Phenylalanine-derived VOCs originate from the catabolism of phenylalanine and include various compounds, such as phenylacetaldehyde, 2-phenylethanol, 1-nitro-2-phenylethane and 2-phenylacetonitrile, significantly contributing to consumer’s liking or disliking, depending on their concentration (Martina et al., 2021). In this pathway, phenylalanine is decarboxylated by aromatic acid decarboxylases (*AADC*s) to convert it to phenethylamine, thus substrate availability is re-directed from the phenylpropanoid pathway (Tieman et al., 2006). Although phenylalanine was over-accumulated in LSL (**Figure 7**), no *AADC* ortholog was differentially expressed between LSL and SSL. This step has been previously demonstrated to be regulated at the transcriptional level (Li et al., 2019). In turn, phenethylamine can generate 1-nitro-2-phenylethane or benzylnitrile by the action of *FMO* using cysteine and the volatile aldehyde phenylacetaldehyde as substrates (Liscombe et al., 2022). In this study, LSL was richer in 1-nitro-2-phenylethane, presumably due to the enhanced accumulation of phenylalanine, offering a musty-earthy odor that has been associated with consumer-liking (Kaur et al., 2023) and regarded as strong umami influencer (Colantonio et al., 2022). At the same time, two *FMO*s were down-regulated in LSL, but these gene copies do not seem to have a key role in phenylalanine-derived VOCs, as previously recorded (Klee and Tieman, 2018). As ethylene can stimulate the catabolism of phenylalanine through the induction of *PAL*s (Klee and Giovannoni, 2011), it is evident that the observed low levels of these metabolites in SSL are strongly under ethylene regulation that leads to the depletion of substrate availability. Over-accumulation of phenylalanine and phenylpropanoid-derived VOCs have been previously correlated with biotic stress responses, due to the up-regulation of the antioxidant defense machinery (Distefano et al., 2022), as well as wild species such as *S. pennellii* (Zhu et al., 2019). Furthermore, phenylalanine-derived VOCs can explain a large fraction of flavor variability within tomato germplasm, largely contributing to sweetness perception (34%) and overall liking score (16%) (Colantonio et al., 2022).

Phenylpropanoid-related (or benzenoids) VOCs consist of a separate group of phenylalanine-derived VOCs, including guaiacol, methyl salicylate, and eugenol, mostly associated from intermediates in the lignin pathway (Zhu et al., 2019). In this pathway, p-coumarate is converted to its CoA ester by *4CL*, in a key branching point of phenylpropanoid metabolism (Cheynier et al., 2013). In LSL, both coumarate content and *4CL* expression were stimulated, probably contributing to the synthesis of methyl salicylate, ethyl salicylate and eugenol (**Figure 7**), which are all considered as major influencers of tomato aroma, and are generally described as medicinal, green, smoky, or pungent odors (Kaur et al., 2023). Among them, eugenol is regarded as unpleasant flavor by consumers due to its pharmaceutical odor and bitter flavor, whereas the concentration of methyl salicylate may determine consumer liking or not (Tieman et al., 2012; Wang et al., 2023). Among the key genes catalyzing the methylation of salicylate, neither salicylic acid methyltransferases nor methyl esterases (Kaur et al., 2023) were differentially expressed between LSL and SSL. By contrast, some transcription factors such as *ETHYLENE INSENSITIVE3* (*EIN3*) that are negative regulators of salicylate (Li et al., 2019) were remarkably induced in SSL (**Supplementary Table 10**), probably accounting for the low levels of both ethyl- and methyl salicylate determined in this study. Usually, these lignin-related compounds are more abundant in unripe fruits, in heterografted plants, in off-season fruits, in biotic stresses, being unwelcomed by consumers (Distefano et al., 2022). This is probably related to their function as endogenous signal molecules to external stimuli.

## 5 Conclusion

This work provides for the first time a complete study of the existing diversity regarding the post-harvest potential of the Greek tomato Gene Bank collection, highlighting the dynamic interactions between ethylene and metabolic pathways that are important in determining tomato fruit quality, nutritional value and aroma. Low ethylene levels in the LSL accession (TRTH1620) were correlated with higher levels of amino acids, carotenoids and phenylpropanoids, as well as with key VOCs of tomato flavor and aroma derived from benzenoid, BCAA and phenylalanine pathways. By contrast, high ethylene levels in the SSL accession (TRTH2510) were associated with high accumulation of tocopherols, and fatty acids. Of outmost importance is the fact that in LSL, a clear shift of the ethylene biosynthetic pathway from methionine towards polyamines is evident, providing interesting clues on the mechanism that regulates fruit ripening and quality attributes in tomato. This mechanism includes the down-regulation of the key ethylene biosynthetic genes (*ACS2*, *ACS4*, *ACO1*) responsible for pre- and post-climacteric ethylene biosynthesis, accompanied by the up-regulation of various *SAMS*s and *SAMDC2*, that re-direct methionine from ethylene biosynthesis towards polyamine metabolism (spermidine). As for ABA-associated pathways, the ABA receptor, *PYL9*, was remarkably repressed in LSL, correlating with the longer shelf-life of TRTH1620. By contrast, *PP2C12*, which acts negatively in ABA signaling regulation, and *CYP707A2* related to ABA catabolism, were both induced in LSL. Although a lot of effort is still required to uncover in detail the exact role of these genes in regulating tomato shelf-life, our findings reinforce the notion that ABA has a central role in fruit postharvest dynamics that needs further exploitation. Among other interesting correlations, ethylene was positively related to cell-wall degrading genes, and AsA accumulation, but it negatively regulated steroidal alkaloids. The observed discrepancies in carotenoid and phenylpropanoid pathways between accumulated metabolites and expressed transcripts clearly points to either a feedback regulation or a post-transcriptional regulation of these routes that merits further attention. Notwithstanding these vibrant differences between genotypes, important VOCs contributing to consumer liking can be found in either of the two genotypes, indicating the complexity of the pathways related to major enhancers of tomato flavor and aroma. Lipid-derived VOCs and the apocarotenoid β-damascenone are mostly accumulated in SSL, whereas LSL was richer in phenylalanine-derived and phenylpropanoid-derived VOCs. Overall, the knowledge generated herein provides a valuable genetic tool for future functional studies that can direct breeding strategies towards the utilization of underutilized germplasm to develop the “perfect” tomato, that can appeal consumer interest on traditional varieties.

## Data availability statement

The datasets presented in this study can be found in online repositories. The names of the repository/repositories and accession number(s) can be found at: https://www.ncbi.nlm.nih.gov/bioproject/PRJNA991925.

## Author contributions

IM: Data curation, Investigation, Methodology, Software, Supervision, Writing – original draft, Writing – review & editing. AK: Data curation, Investigation, Methodology, Writing – original draft, Writing – review & editing. SF: Methodology, Writing – review & editing. JR: Methodology, Writing – review & editing. EP: Data curation, Methodology, Writing – review & editing. SN: Data curation, Methodology, Writing – review & editing. CP: Data curation, Methodology, Writing – review & editing. SK: Methodology, Writing – review & editing. KN: Methodology, Writing – review & editing. AG: Methodology, Writing – review & editing. GD: Methodology, Writing – review & editing. AKK: Conceptualization, Funding acquisition, Investigation, Project administration, Resources, Supervision, Writing – original draft, Writing – review & editing.
