# Supplementary material for: A metabolome and transcriptome survey to tap the dynamics of fruit prolonged shelf-life and improved quality within Greek tomato germplasm
Source: Front Plant Sci. 2023 Sep 25;14:1267340. doi: 10.3389/fpls.2023.1267340 (PMC10560995; doi:10.3389/fpls.2023.1267340)
Supplement: Supplementary file 1 [file DataSheet_1.pdf]

Supplementary Material

1 Supplementary Data

Supplementary Table 1. List of primers for RT-qPCR validation.

Supplementary Table 2. Analysis of variance (ANOVA) and descriptive statistics of 130 Greek tomato accessions for harvested fruit at breaker stage and their postharvest ripening characteristics. CI: Color Index change; CS: cold storage period; FLR: firmness loose rate; SL: during shelf life.

Supplementary Table 3. Metabolomic profiles of the seven SSL and the seven LSL genotypes, with regard to different pathways related to phytonutrients and volatile compounds.

Supplementary Table 4. Statistical analysis of metabolites determined in the seven SSL and the seven LSL based on Student's t-test.

Supplementary Table 5. Pearson correlation coefficient for the 36 phytonutrients compounds and the 60 VOCs within out sample set of 14 tomato accessions.

Supplementary Table 6. Differentially accumulated metabolites over-accumulated in LSL (TRTH1620; red) or in SSL (TRTH2510; green), with $p < 0.05$.

Supplementary Table 7. Mapping statistics of RNASeq.

Supplementary Table 8. FPKM values above 1 in either of the two genotypes.

Supplementary Table 9. Differentially expressed metabolites (DEGs) found between TRTH2510 (SSL) and TRTH1620 (LSL), based on $|\log2(FC) > 2|$ and corrected (p values ≤ 0.01).

Supplementary Table 10. Selected differentially expressed metabolites (DEGs) found between TRTH2510 (SSL) and TRTH1620 (LSL) from key metabolic pathways related to postharvest potential, hormones, amino acids, fatty acids, secondary metabolism and volatiles, based on p-values ≤ 0.01.

2 Supplementary Figures and Tables

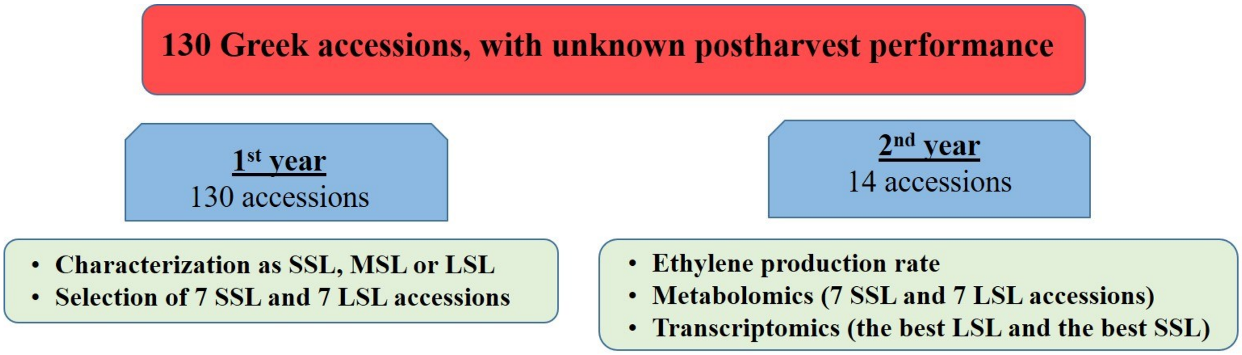

Supplementary Figure 1. Graphical abstract of the experimental design.

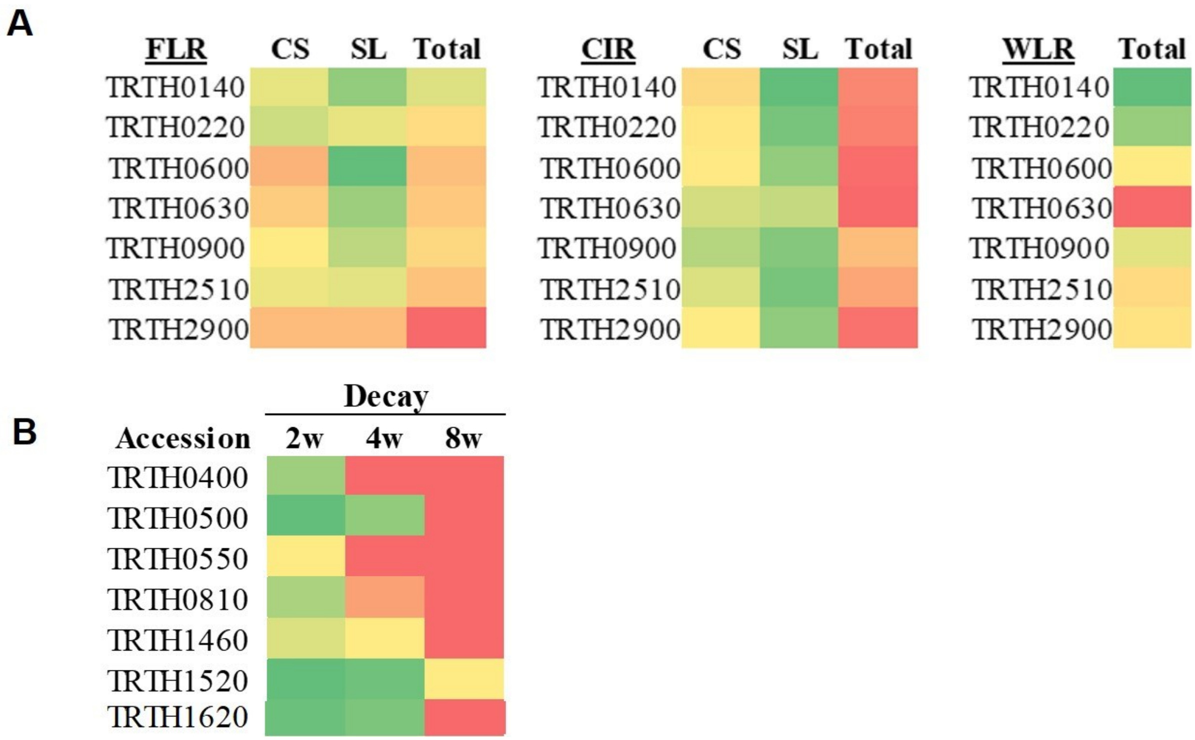

Supplementary Figure 2. (A) Heat map of seven (7) SSL accessions based on their firmness loose rate (FLR), color index rate (CIR) and weight loose rate (WLR) during storage stored at 12 ºC for 10 days plus 20 ºC for 3 days. (B) Heat map of seven (7) LSL accessions based on their decay rate during storage at 20 ºC. Red color indicates high values, and green color low values.

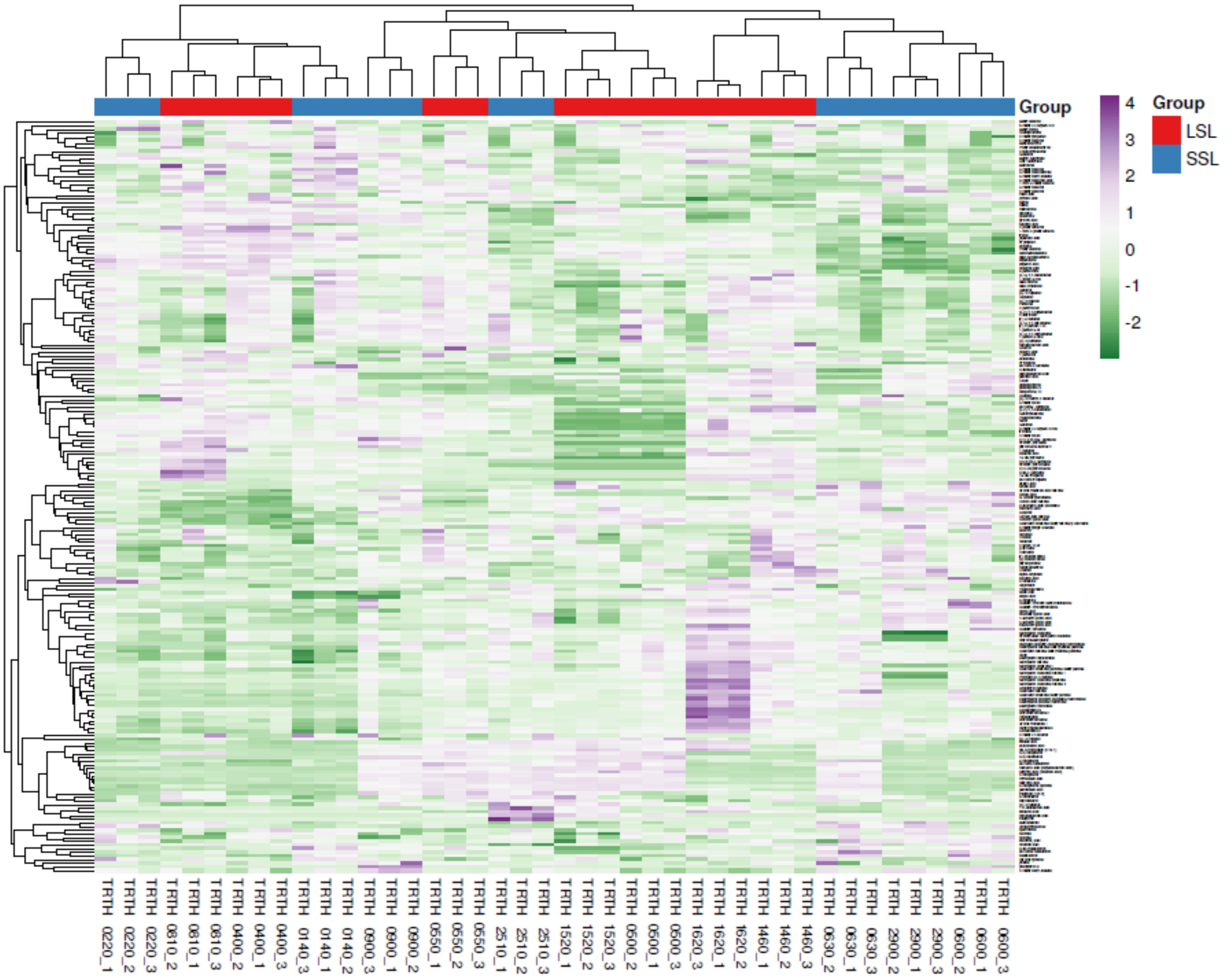

Supplementary Figure 3. Heatmap of the 206 metabolites that were present in tomato fruits for each variety, with three replications each one. Data are log transformed.

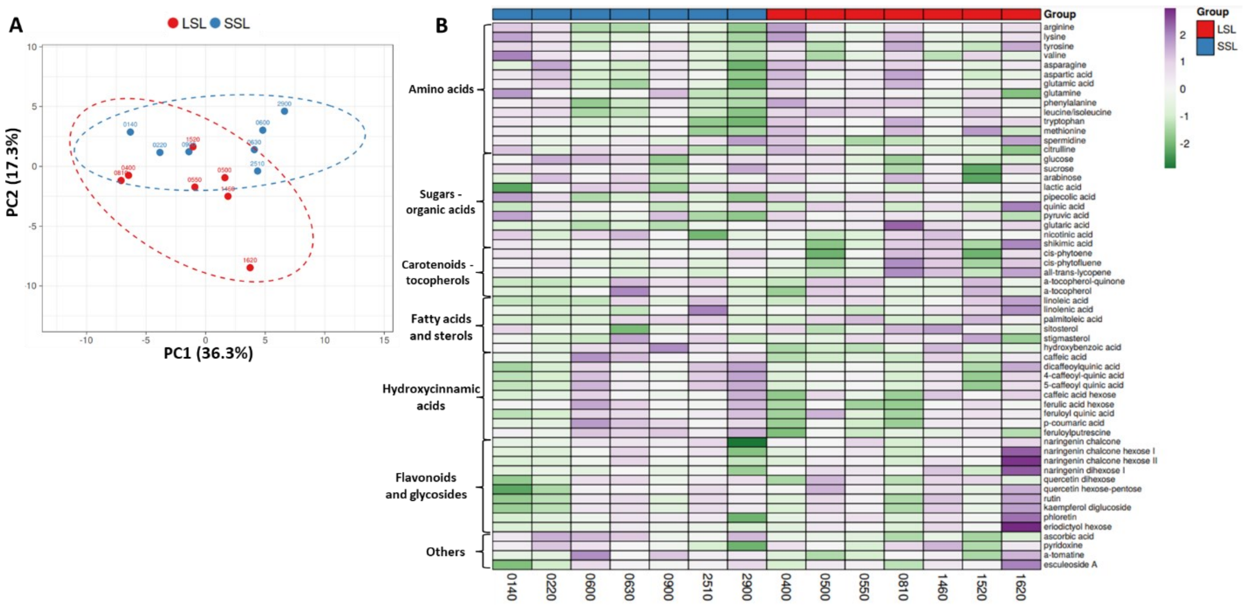

Supplementary Figure 4. Metabolome analysis of phytonutrients in tomato fruits from seven SSL and seven LSL varieties. (A) PCA of samples with three biological replicates for each variety. (B) Heatmap of different phytonutrients that were present in tomato fruits for each variety. Data are log transformed.

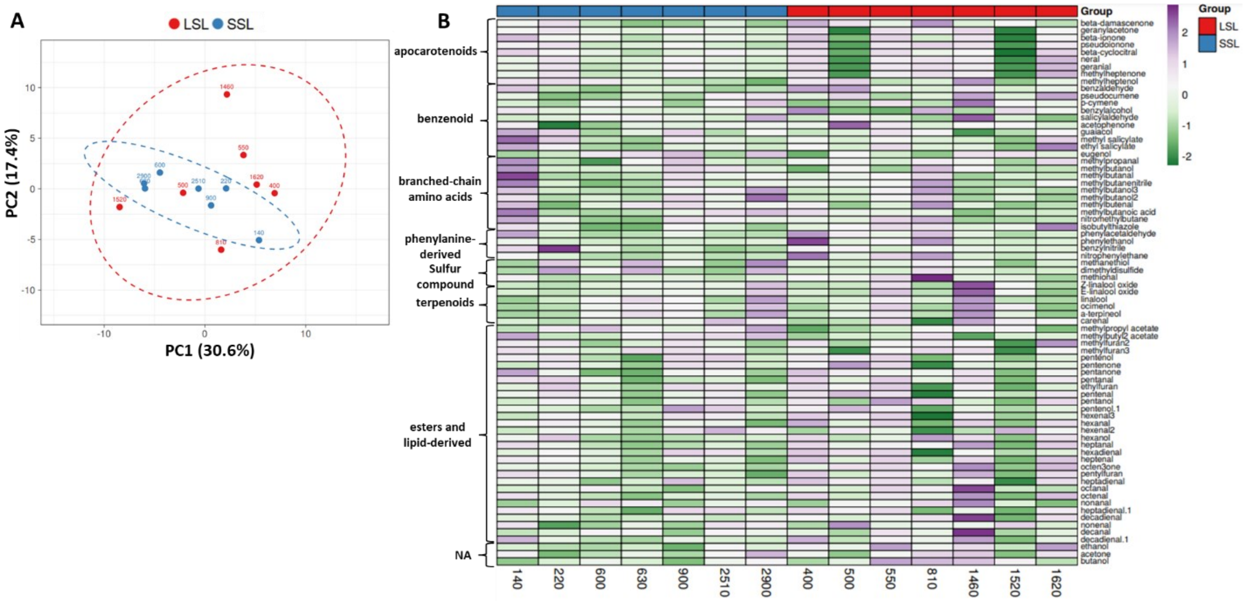

Supplementary Figure 5. Metabolome analysis of VOCs in tomato fruits from seven SSL and seven LSL varieties. (A) PCA of samples with three biological replicates for each variety. (B) Heatmap of different VOCs that were determined in tomato fruits for each variety. Data are log transformed.

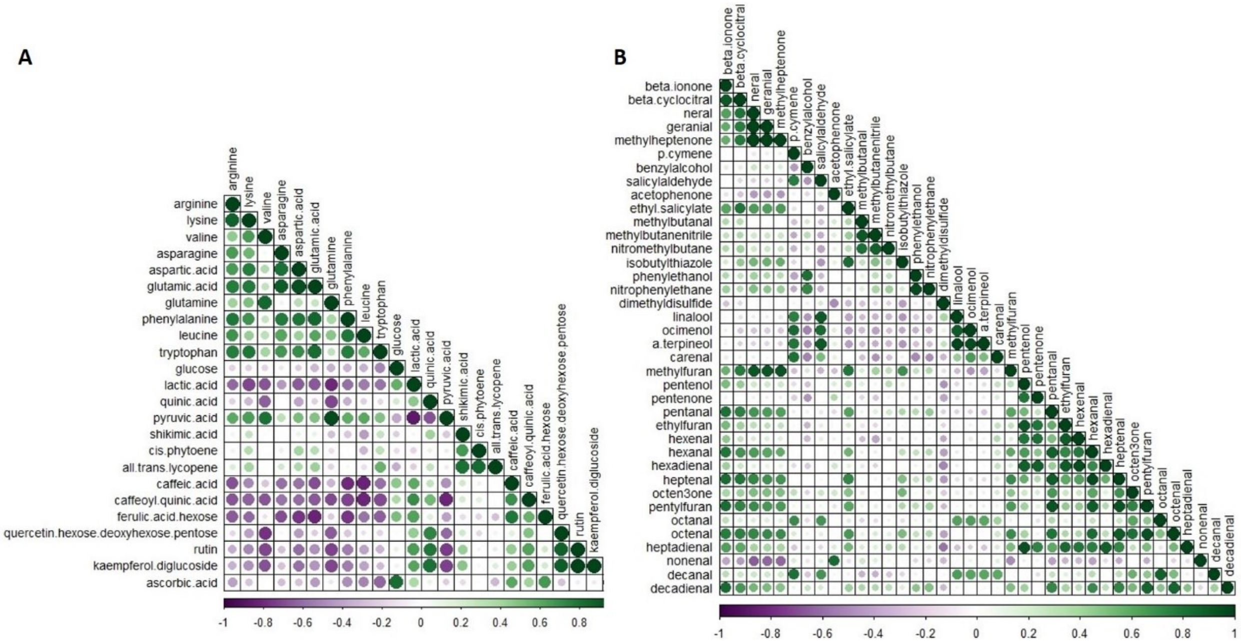

Supplementary Figure 6. Pearson correlation of selected phytonutrients (A) and VOCs (B) with significant correlations ($p<0.05$ and $R^2 >0.8$).

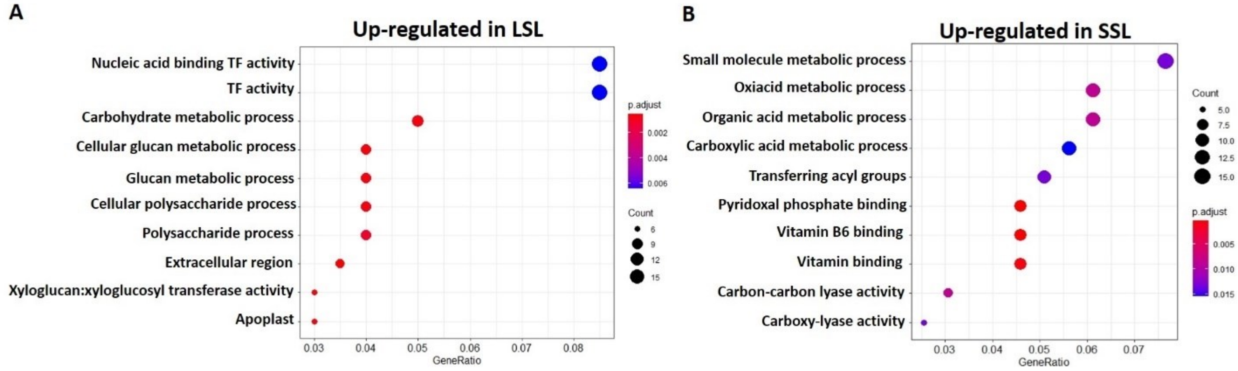

Supplementary Figure 7. Dot-plot of GO annotation enrichment of DEGs up-regulated in the LSL genotype (TRTH1620; A) or in the SSL genotype (TRTH2510; B).

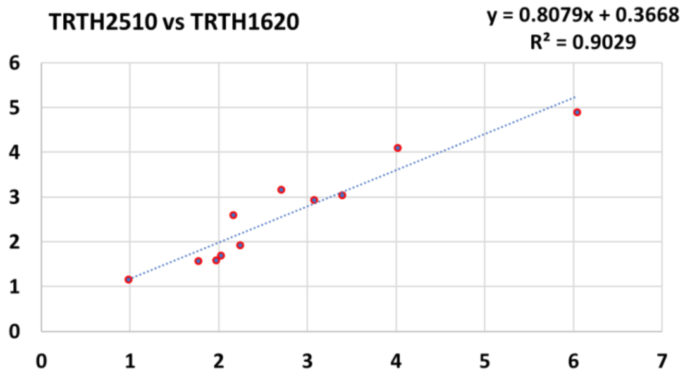

Supplementary Figure 8. Comparison between Log2 fold change (FC) from qRT-PCR values and Log2 FC from RNA-seq of the selected DEGs between TRTH2510 (SSL) and TRTH1620 (LSL). Three biological replicates were employed for both datasets. Relative expression was normalized against CAC, while primers are reported in Supplementary Table 1.

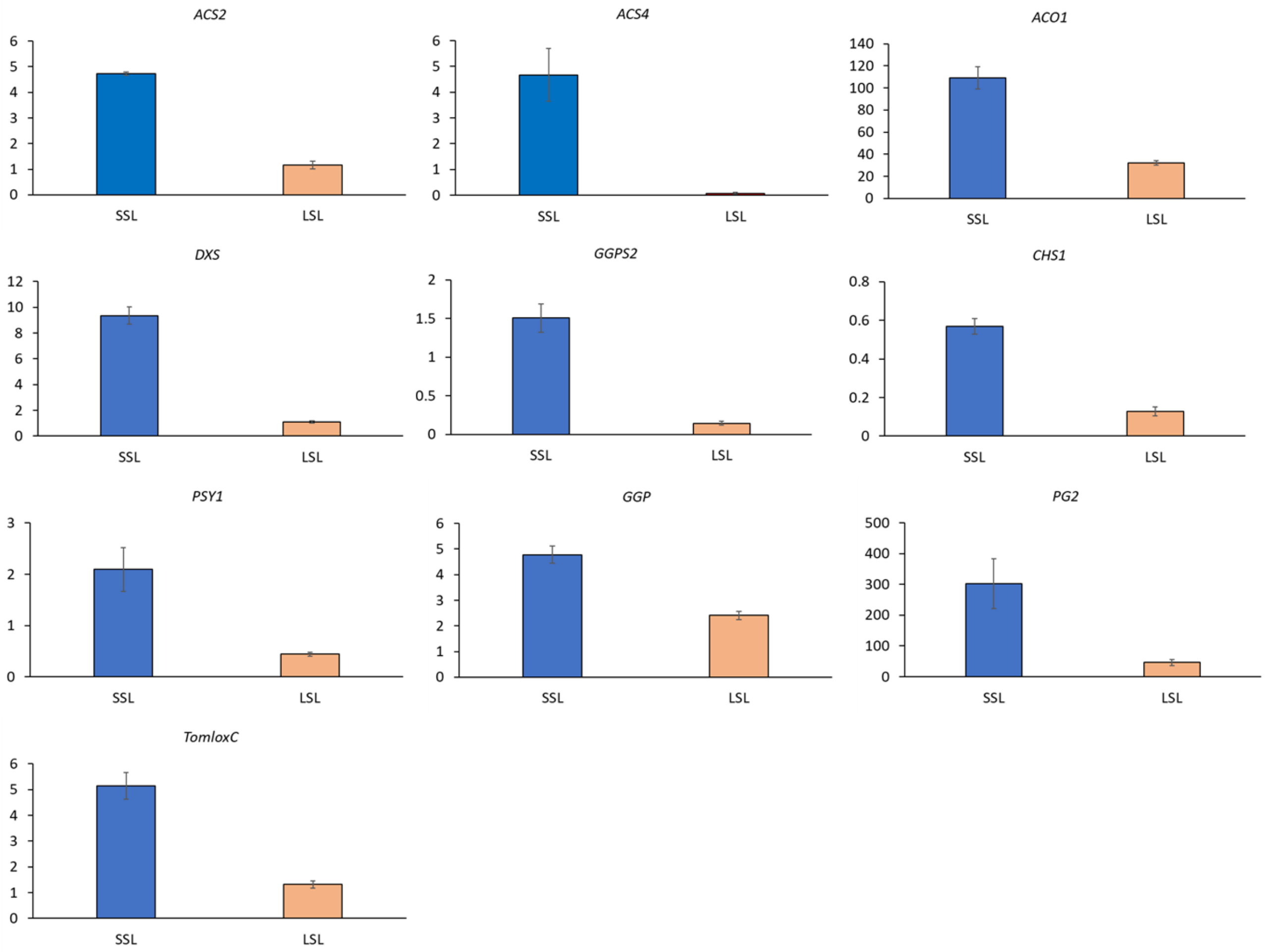

Supplementary Figure 9. Relative expression of ACS2, ACS4, ACO1, DXS, GGPS2, CHS1, PSY1, GGP, PG2 and TomloxC based on qRT-PCR analysis between TRTH2510 (SSL) and TRTH1620 (LSL). Three biological replicates were employed for both datasets. Relative expression was normalized against CAC, while primers are reported in Supplementary Table 1.
